# Supplementary material for: Challenges implementing a carer support intervention within a national stroke organisation: findings from the process evaluation of the OSCARSS trial
Source: BMJ Open. 2021 Jan 12;11(1):e038129. doi: 10.1136/bmjopen-2020-038129 (PMC7805355; doi:10.1136/bmjopen-2020-038129)
Supplement: Supplementary data [file bmjopen-2020-038129supp001.pdf]

SUPPLEMENTARY MATERIAL 1

Topic guides for carer interviews

First interviews: Questions about experiences of support

I'd like to speak to you about contact you've had from [stroke organisation].

- Can you talk me through what happened during the visit?
    - Did you talk with X on your own or with others? (how was that / how did that go?)
    - Was any paperwork used? Possible additional probs : Can you tell me more about the paperwork you used? How was that / how did that go?
    - Did you keep a copy of any paperwork? If so, do you ever refer to it / find it helpful?
    - Did you talk about any support that you might need , as someone who is providing care/ support to <INSERT RELATIONSHIP eg mum >> How was that / How did that go?
    - Did you feel that you or the staff member 'took the lead' when talking? (Could re-visit 'paperwork' prompts here too)
- Did you come up with ideas of how to address the needs/issues you talked about? Can you talk me through them ?
    - Depending on if/how addressed earlier could repeat: Did you feel that you or the coordinator 'took the lead' when coming up with ideas?
    - Were you happy with the solutions/ ideas for support?
    - Were there any things that are not being addressed/ that it wasn't possible to address? What were these? How do you feel about this?
- Do you have any plans to talk with (name) / the stroke organisation again about support for you?
- What kind of support did you expect when you accepted a [stroke organisation] visit?
- Did it meet your expectations?
- Overall, what were your impressions of the visit?
- Are there any practical ways that the [stroke organisation] has influenced your own life as a caregiver? [Or have you found your own means of support, without reference to the stroke organisation?]

Second interviews: Questions about perceptions of impact support

[Last time we spoke] You told me about the things that were suggested that might be of help. Did you try any of these things?

- If no, why not?

If yes :

- Did they help? Have you continued to use this support?
    - Why/why not?
    - Has it lead to other things that have helped you as a carer?
- Is there any support you would have liked but felt like you didn't get?
    - Why? / how do you think it would have helped?
    - Has that changed over time?

- Overall - How did you find the [stroke organisation] in terms of supporting you as a carer? [Was it helpful/unhelpful?] Explore more:
    - Did it make any difference to you as a carer? If yes - in what way?
    - Has the support you received made any difference to the person you care for?
    - Is there anything you would have liked to have been done differently?

Finally

- Is there anything else you would like to tell us about the support you received / support for carers that we haven’t covered?
