# Supplementary material for: Challenges implementing a carer support intervention within a national stroke organisation: findings from the process evaluation of the OSCARSS trial
Source: BMJ Open. 2021 Jan 12;11(1):e038129. doi: 10.1136/bmjopen-2020-038129 (PMC7805355; doi:10.1136/bmjopen-2020-038129)
Supplement: Supplementary data [file bmjopen-2020-038129supp002.pdf]

SUPPLEMENTARY MATERIAL 2

Topic guides for staff

Frontline staff topic guide

Current practice

- Could you tell me briefly about your role [core tasks/expectations] within the stroke organisation? (how long?)
    - Could you tell me about your role in relation to supporting carers?
- Could you talk me through your current process of supporting carers?
    - Identifying (and defining) carers
    - Initial contact (face to face/ by phone; planned or unplanned? Always with stroke survivor or alone – and how does this happen?)
    - Identifying needs (paperwork; recording information? Action plan / follow up?)
    - Are there any other things that help you support carers? (e.g. local services)
    - Anything that prevents you from supporting carers?
- How have you found the OSCARSS recruitment process?

Changes to current practice

- Apart from OSCARSS are there any other factors that have impacted on how you support carers?
    - Has the CSNAT-Stroke been discussed within or/and across services? (e.g. through networking/learning events) – **control arm, skip to final question**

Intervention arm only:

- Have you been using CSNAT-Stroke during your visits that have involved carers? (If no – reasons why not? If yes – how did you use it? How have you found using it?)
- How would you describe CSNAT-Stroke?
- Similarities or differences of CSNAT-Stroke to how you've worked with carers before? If no difference – why is this?
- Any occasions where you would use an alternative process to address carer needs?
    - Any adaptation of CSNAT-Stroke or deliberately doing something else? Why?

Mechanisms that have supported changes

- Can you tell me about anything that has supported your use and understanding of CSNAT-Stroke
- Has there been anything that has hindered your use of CSNAT-Stroke? What could have helped overcome these hindrances?

Outcomes

- Through using CSNAT-Stroke do you feel like this has had any impact on your work with carers? Any impact on your work with stroke survivors?
- Do you think you would you continue to follow CSNAT-Stroke in your work with carers (why/why not)? What would you need to support the continued use of CSNAT-Stroke (on an individual and organisational level)?
- What is your overall opinion of CSNAT-Stroke?
- Is there anything else you would like to tell us about how you support carers that we haven't covered?

Managers and Senior Leaders: Topic guide

Current practice

- Could you tell me about your role within the organisation? (Involvement in supporting carers/ knowledge of how carers are supported?)
- How important do you feel supporting carers is within the organisation?
- What is needed to provide carer support? Who in the organisation should provide carer support?

Changes to current practice

- How much do you know about CSNAT-Stroke? (similarities or differences to standard practice etc.)
- How did you find out about CSNAT-Stroke?

Mechanisms that have supported changes

- Do you know of anything that has helped or hindered the use of CSNAT-Stroke?
- What structures would need to be in place to support the use of an approach like CSNAT-Stroke?

Outcomes

- Has the use of CSNAT-Stroke within the organisation created any changes?
    - Anything else that might have contributed to these changes?
- Do you think that the use of CSNAT-Stroke will be continued to support carers (why/why not)?
    - Does it feel like part of the organisation’s approach to supporting carers now?
    - What would the organisation need to support the continued use of CSNAT-Stroke?
    - Who would be best placed/resourced to provide the training for CSNAT-Stroke?
- What is your overall opinion of CSNAT-Stroke?
- Is there anything else you would like to tell us about how carers are supported or CSNAT-Stroke that we haven’t covered?
