# Supplementary material for: Challenges implementing a carer support intervention within a national stroke organisation: findings from the process evaluation of the OSCARSS trial
Source: BMJ Open. 2021 Jan 12;11(1):e038129. doi: 10.1136/bmjopen-2020-038129 (PMC7805355; doi:10.1136/bmjopen-2020-038129)
Supplement: Supplementary data [file bmjopen-2020-038129supp003.pdf]

**SUPPLEMENTARY MATERIAL 3**

**T2 and T3 questionnaire items based on NoMAD for frontline staff in the intervention arm only**

| | T2 questionnaire (N=20) | | | T3 questionnaire (N=20) | | |
|---|---|---|---|---|---|---|
| **Item** | **Agree** ***n*** | **Neutral** ***n*** | **Disagree** ***n*** | **Agree** ***n*** | **Neutral** ***n*** | **Disagree** ***n*** |
| **Coherence** | | | | | | |
| I can see how the CSNAT Stroke differs from usual ways of working | 14 | 4 | 2 | 11 | 8 | 1 |
| Staff in this service have a shared understanding of the purpose of the CSNAT-Stroke | 13 | 7 | 0 | 10 | 7 | 3 |
| I can see the potential value of the CSNAT-Stroke for my work | 17 | 0 | 3 | 18 | 2 | 0 |
| I understand how the CSNAT-Stroke affects the nature of my own work | 18 | 2 | 0 | 17 | 2 | 1 |
| **Cognitive participation** | | | | | | |
| I believe that participating in the CSNAT-Stroke is a legitimate part of my role | 15 | 3 | 2 | 16 | 4 | 0 |
| There are key people in the Stroke Association who drive the CSNAT Stroke forward and get others involved | 5 | 11 | 4 | 5 | 12 | 3 |
| I'm open to working with colleagues in new ways to use the CSNAT-Stroke | 16 | 2 | 2 | 16 | 4 | 0 |
| **Collective action** | | | | | | |
| I can easily integrate the CSNAT-Stroke into my working practice. | 14 | 1 | 5 | 13 | 6 | 1 |
| Sufficient resources are available to support the CSNAT Stroke | 11 | 3 | 6 | 13 | 3 | 4 |
| Sufficient training is provided to enable staff in this service to implement the CSNAT-Stroke | 14 | 2 | 4 | 17 | 3 | 0 |
| I have confidence in other people's ability to use the CSNAT-Stroke | 10 | 10 | 0 | 11 | 9 | 0 |
| Work is assigned to those with skills appropriate to the CSNAT-Stroke | 5 | 13 | 2 | 7 | 12 | 1 |
| Management adequately supports the CSNAT-Stroke | 1 | 13 | 6 | 10 | 7 | 3 |
| **Reflexive monitoring** | | | | | | |
| Staff in this service agree that the CSNAT-Stroke is worthwhile | 13 | 5 | 2 | 13 | 7 | 0 |
| I value the effects that the CSNAT-Stroke has had on my work | 12 | 5 | 3 | 15 | 5 | 0 |
| Feedback about the CSNAT-Stroke can be used to improve it in the future | 16 | 4 | 0 | 20 | 0 | 0 |

| | | | | | | |
|---|---|---|---|---|---|---|
| I can modify how I work with the CSNAT-Stroke | 16 | 1 | 3 | 13 | 6 | 1 |
