# Supplementary material for: Challenges implementing a carer support intervention within a national stroke organisation: findings from the process evaluation of the OSCARSS trial
Source: BMJ Open. 2021 Jan 12;11(1):e038129. doi: 10.1136/bmjopen-2020-038129 (PMC7805355; doi:10.1136/bmjopen-2020-038129)
Supplement: Author's manuscript [file bmjopen-2020-038129.draft_revisions.pdf]

BMJ Open

BMJ Open is committed to open peer review. As part of this commitment we make the peer review history of every article we publish publicly available.

When an article is published we post the peer reviewers' comments and the authors' responses online. We also post the versions of the paper that were used during peer review. These are the versions that the peer review comments apply to.

The versions of the paper that follow are the versions that were submitted during the peer review process. They are not the versions of record or the final published versions. They should not be cited or distributed as the published version of this manuscript.

BMJ Open is an open access journal and the full, final, typeset and author-corrected version of record of the manuscript is available on our site with no access controls, subscription charges or pay-per-view fees (http://bmjopen.bmj.com).

If you have any questions on BMJ Open's open peer review process please email info.bmjopen@bmj.com

BMJ Open

CHALLENGES IMPLEMENTING A CARER SUPPORT INTERVENTION WITHIN A NATIONAL STROKE ORGANISATION: FINDINGS FROM THE PROCESS EVALUATION OF THE OSCARSS TRIAL

Journal:	*BMJ Open*
Manuscript ID	bmjopen-2020-038129
Article Type:	Original research
Date Submitted by the Author:	28-Feb-2020

Complete List of Authors:	Darley, Sarah; The University of Manchester, Alliance Manchester Business School<br>Knowles, Sarah; The University of Manchester, Alliance Manchester Business School<br>Woodward-Nutt, Kate; Salford Royal Hospitals NHS Trust<br>Mitchell, Claire; The University of Manchester, Manchester Academic Health Sciences Centre (MAHSC), Division of Neuroscience and Experimental Psychology<br>Grande, Gunn; The University of Manchester, Manchester Academic Health Sciences Centre (MAHSC), Division of Nursing, Midwifery and Social Work<br>Ewing, Gail; University of Cambridge, Centre for Family Research<br>Rhodes, Sarah; The University of Manchester, Manchester Academic Health Sciences Centre (MAHSC), Centre for Biostatistics, Institute of Population Health<br>Bowen, Audrey; The University of Manchester, Manchester Academic Health Sciences Centre (MAHSC), Division of Neuroscience and Experimental Psychology<br>Patchwood, Emma; The University of Manchester, Manchester Academic Health Sciences Centre (MAHSC), Division of Neuroscience and Experimental Psychology

Keywords:	Stroke < NEUROLOGY, QUALITATIVE RESEARCH, PUBLIC HEALTH

SCHOLARONE™ Manuscripts

I, the Submitting Author has the right to grant and does grant on behalf of all authors of the Work (as defined in the below author licence), an exclusive licence and/or a non-exclusive licence for contributions from authors who are: i) UK Crown employees; ii) where BMJ has agreed a CC-BY licence shall apply, and/or iii) in accordance with the terms applicable for US Federal Government officers or employees acting as part of their official duties; on a worldwide, perpetual, irrevocable, royalty-free basis to BMJ Publishing Group Ltd ("BMJ") its licensees and where the relevant Journal is co-owned by BMJ to the co-owners of the Journal, to publish the Work in this journal and any other BMJ products and to exploit all rights, as set out in our licence.

The Submitting Author accepts and understands that any supply made under these terms is made by BMJ to the Submitting Author unless you are acting as an employee on behalf of your employer or a postgraduate student of an affiliated institution which is paying any applicable article publishing charge ("APC") for Open Access articles. Where the Submitting Author wishes to make the Work available on an Open Access basis (and intends to pay the relevant APC), the terms of reuse of such Open Access shall be governed by a Creative Commons licence – details of these licences and which Creative Commons licence will apply to this Work are set out in our licence referred to above.

Other than as permitted in any relevant BMJ Author's Self Archiving Policies, I confirm this Work has not been accepted for publication elsewhere, is not being considered for publication elsewhere and does not duplicate material already published. I confirm all authors consent to publication of this Work and authorise the granting of this licence.

For peer review only

TITLE PAGE

CHALLENGES IMPLEMENTING A CARER SUPPORT INTERVENTION WITHIN A NATIONAL STROKE ORGANISATION: FINDINGS FROM THE PROCESS EVALUATION OF THE OSCARSS TRIAL

Corresponding author
Name: Dr Sarah Darley
Address: Suite 1 Williamson Building, Centre for Primary Care and Health Services Research, Faculty of Biology, Medicine and Health, University of Manchester, Oxford Road, Manchester M13 9QQ, UK
Email: Sarah.Darley@manchester.ac.uk
Telephone: +44 161 275 7687

Authors: Sarah Darley, Sarah Knowles, Kate Woodward-Nutt, Claire Mitchell, Gunn Grande, Gail Ewing, Sarah Rhodes, Audrey Bowen, and Emma Patchwood on behalf of the OSCARSS Research Team

Author Affiliations

University of Manchester, Alliance Manchester Business School: Sarah Knowles PhD, Sarah Darley PhD

University of Manchester, Manchester Academic Health Sciences Centre (MAHSC), Manchester, UK:

- Division of Neuroscience and Experimental Psychology, School of Biological Sciences: Emma Patchwood PhD, Prof Audrey Bowen PhD, Claire Mitchell, PhD
- Division of Nursing, Midwifery and Social Work, School of Health Sciences: Prof Gunn Grande PhD
- Centre for Biostatistics, Institute of Population Health: Sarah Rhodes MSc

University of Cambridge, Centre for Family Research, Cambridge, UK: Gail Ewing PhD

Salford Royal Foundation Trust, Salford, UK: Kate Woodward-Nutt MSc

Manuscript word count (excluding title page, abstract, references, figures and tables): 4152
Abstract word count: 284
Tables: 3
Figures: 1

For peer review only

KEYWORDS
Stroke, Caregivers, Normalisation Process Theory, Process evaluation

ABSTRACT

Objectives
To examine the implementation of an intervention to support informal caregivers and to help understand findings from the Organising Support for Carers of Stroke Survivors (OSCARSS) cluster randomised controlled trial (cRCT).

Design
Longitudinal process evaluation using mixed methods. Normalisation Process Theory informed data collection and provided a sensitising framework for analysis.

Setting
Specialist stroke support services delivered primarily in the homes of informal carers of stroke survivors.

Participants
OSCARSS cRCT participants including carers, organisational staff, managers and senior leaders.

Intervention
The Carer Support Needs Assessment Tool for Stroke (CSNAT-Stroke) intervention is a staff-facilitated, carer-led approach to help identify, prioritise, and address support needs.

Results
We conducted qualitative interviews with: OSCARSS cRCT carer participants (11 intervention, 10 control), staff (12 intervention, 8 control), and managers and senior leaders (11); and obtained 140 responses to an online staff survey over three separate time points. Both individual (carer/staff) and organisational factors impacted implementation of the CSNAT-Stroke intervention and how it was received by carers. Staff valued the idea of a structured approach to supporting carers, but key elements of the intervention were not routinely delivered. Carers did not necessarily identify as "carers", which made it difficult for staff to engage them in the intervention. Despite organisational enthusiasm for OSCARSS, staff in the intervention arm perceived support and training for implementation of CSNAT-Stroke as delivered primarily by the research team, with few opportunities for shared learning across the organisation.

Conclusions
We identified challenges across carer, staff, and organisation levels that help explain the OSCARSS cRCT outcome. Ensuring training is translated into practice and ongoing organisational support would be required for full implementation of this type of intervention, with emphasis on the carer-led aspects, including supporting carer self-identification.

Trial registration
OSCARSS cRCT ISRCTN58414120

ARTICLE SUMMARY

Strengths and limitations of this study

- This study demonstrates the value of an embedded process evaluation for enriching understanding of neutral findings from a trial of support for carers of stroke survivors.
- The longitudinal study used mixed methods to triangulate data and incorporate findings from a wide range of perspectives, including staff and carers from both arms of the study as well as the organisational context.
- Intervention fidelity was difficult to assess without real-time observation but qualitative findings guided by Normalisation Process Theory captured challenges of implementing a carer-led approach.

FUNDING STATEMENT

This project was supported by the National Institute for Health Research Collaboration for Leadership in Applied Health Research and Care (NIHR CLAHRC) Greater Manchester and Stroke Association. NIHR had no role in study design, data collection, data analysis, data interpretation, or writing of the paper. Stroke Association partnered with NIHR in funding this study and was the specialist stroke service provider in OSCARSS. Some

For peer review only

Stroke Association staff were research participants in this study and others contributed to discussions about findings and dissemination. The first author had full access to all the study data and had final responsibility for the decision to submit for publication. The views expressed in this article are those of the author(s) and not necessarily those of the NIHR, the Department of Health and Social Care, or the Stroke Association.

COMPETING INTERESTS

Dr. Grande reports grants from NIHR CLAHRC, during the conduct of the study; In addition, Dr. Grande has a patent Copyright issued.
Dr. Ewing reports grants from NIHR, during the conduct of the study; In addition, Dr. Ewing has a patent Copyright issued.
Mrs. Rhodes reports grants from NIHR, during the conduct of the study.
Prof. Bowen reports grants from NIHR, grants from Stroke Association, during the conduct of the study; grants from Stroke Association, grants from NIHR, outside the submitted work.
Dr. Patchwood reports grants from Stroke Association, outside the submitted work.
Dr Darley, Dr Knowles, Ms Woodward-Nutt and Dr Mitchell have nothing to disclose.

INTRODUCTION

Background

Informal caregivers, such as family members, play a significant role in providing daily support to stroke survivors who experience a range of life-limiting difficulties [1]. The caring role can be rewarding but is also challenging with significant health implications [2], impacting on physical and emotional well-being, financial security, professional and social activities [3]. Current support for carers differs according to location but generally comes from hospital stroke units, local authority, specialist carer organisations, and stroke organisations.

Supporting informal carers is a statutory priority [4, 5, 6] but there is little clarity on how best to identify and assess the needs of people caring informally for stroke survivors. One relevant approach is the Carer Support Needs Assessment Tool (CSNAT) intervention [7]: a comprehensive evidence-based assessment tool integrated within a multi-stage person-centred approach to individualised support. It was developed, implemented and tested in the context of palliative care with positive outcomes [8, 9, 10]. For the present study, the CSNAT intervention was adapted for use in stroke care, through collaboration with a specialist stroke organisation and a group of lay carers. The adaptation resulted in the CSNAT-Stroke intervention, including a staff training and implementation package tailored to the stroke organisation. In brief, the intervention is a person-centred, structured process of needs assessment and support that is practitioner-facilitated, but carer-led, comprised of a four-stage process, including a needs assessment tool and action plan (see Table 1).

Table 1 CSNAT-Stroke intervention as intended in OSCARSS

Key details of the intervention using descriptors from the TIDieR checklist [28], adapted from Patchwood et al. [paper submitted]

Item	CSNAT-Stroke intervention, as intended for implementation within the stroke organisation
Why	To provide a person-centred process of assessment and support for carers that is practitioner-facilitated but carer-led and tailored to the carer's individual needs, which are likely to change over time. The intervention assumes that carers may have difficulty considering and expressing their needs.
What (materials and procedures)	Materials / procedures include: (A) four-stage process (components outlined below), (B) a needs assessment tool, (C) an action plan.<br><br>**1. Identifying and introducing:**<br><br>Staff identify the carer early and make clear that support is available ('scripts' for sensitive use of the term "carer"). Staff introduce needs assessment tool to carers during separate time with the carer, providing an opportunity to discuss support needs.<br><br>**2. Needs assessment**<br><br>Carers are given time to consider their support needs, self-completing the tool, identifying the areas in which they need more support and prioritising those most important to them. Staff

For peer review only

	normalise the practice of having separate time with the carer as well as stroke survivor if present, to support each as individuals.<br>**3. Assessment conversation and tailoring**<br>An assessment conversation between carer and stroke practitioners identifies the carer's individual support needs and what they feel would be most supportive, within the domains of the needs assessment tool that they have prioritised. Support may be directly delivered by staff at this time (e.g. active listening, information, signposting/referrals) but helping carers identify sources of support they may wish to access themselves (self-help) or via family members and friends is also encouraged. Staff create service directories to support signposting/referral.<br>**4. Shared plan for action and review**<br>Carers record a plan of supportive input describing the actions taken or to be taken by the practitioner or carer to address identified needs, which will be subsequently reviewed as appropriate.<br>Each stage should be staff-facilitated but carer-led. At all stages the carer should be given the opportunity to say what is most important to them and what they feel would help support them.
Who provided	Frontline staff: Essential criteria for recruitment to this role includes GCSE education, experience providing care to vulnerable people, and good communication skills. Training in stroke and stroke-specific care is provided by the organisation. Additional intervention specific training provided to frontline staff in clusters allocated to intervention.<br>Training, co-delivered by the service provider and the research team, is with groups of staff over a half-day session involving instructional videos and scripts, role-play, and workbook completion.
How	After training the intervention should be implemented by staff during their routine support visits.
Where	Typically in carers' homes.
When and How much	The intervention should be used every time a staff member has contact with a carer and requires a minimum of 1 face-to-face support contact with the carer, with reviews likely.
Tailoring and modifications	Staff training is modified for those joining after the primary roll-out of the intervention and adapted to one-to-one delivery by the research team using video conferencing.

Organising Support for Carers of Stroke Survivors (OSCARSS) was a cluster randomised controlled trial (cRCT) which aimed to investigate the clinical- and cost-effectiveness of the CSNAT-Stroke intervention for supporting carers relative to a control of usual practice within the stroke organisation [11]. OSCARSS found no meaningful differences in self-reported clinical outcomes for carers, such as strain and distress, between intervention and control groups [paper submitted]. This paper presents findings from the embedded process evaluation of OSCARSS, which aimed to enrich understanding of the trial result, understand the factors influencing implementation and acceptability of a carer-led intervention, and consider implications for future research and practice.

METHODOLOGICAL APPROACH

Process evaluations provide insight into the 'black box' of implementation of a complex intervention [12] and help explain discrepancies between expected and observed outcomes within a context [13]. The process evaluation for OSCARSS used longitudinal mixed methods to examine ongoing experiences of and perspectives on both usual practice and the CSNAT-Stroke intervention from staff within the stroke organisation, as well as carers who receive their support and consented to join OSCARSS.

Normalisation Process Theory (NPT) was used as a sensitising framework for both data collection and analysis. NPT is a commonly used framework in process evaluations of complex interventions to explain the success or failure of implementation projects [14], considering both agency (individual factors) and context (organisational

For peer review only

factors). NPT proposes four constructs to explain how interventions are embedded into practice: Coherence (sense-making), Cognitive Participation (engagement and enrollment), Collective Action (how the work happens in existing systems), and Reflexive Monitoring (evaluation)[15].

PATIENT, CARER, AND PUBLIC INVOLVEMENT (PCPI)

A study specific Research User Group (RUG) of ten individuals with experience of caring for a stroke survivor was set up in December 2015, at the planning stages of OSCARSS [paper submitted]. Through regular meetings (2015-2019) and representation on the Trial Management Group the priorities, experiences, and preferences of the RUG informed development of the research questions and the design, analysis, and dissemination of both the trial and process evaluation. The RUG advised on participant recruitment and were central in limiting the burden of participation for carers. The RUG also supported adaptation of the CSNAT intervention and staff training package, including role-playing videos of the intervention in practice. A video summarising their role in OSCARSS is available on the study website: https://www.arc-gm.nihr.ac.uk/projects/oscarss In the early stages of data analysis three meetings were held to focus on the process evaluation where SD, SK and EP shared and discussed themes from the data with the RUG. In these meetings, NPT and the value of this framework for understanding the findings were collaboratively discussed. The RUG helped us consider rival explanations, new ideas, and implications on practice.

SETTING AND SAMPLE

The OSCARSS cRCT ran from January 2017 to December 2018. It recruited 414 carers over an 18 month recruitment period from 35 randomised clusters made up of the stroke organisation's services across England and Northern Ireland (18 intervention and 17 control). Approximately 100 frontline staff who were employed at participating clusters during the study period were trained to participate in OSCARSS.

The process evaluation purposefully sampled a wide range of participating frontline cluster staff and consented carers from both trial arms. Staff, managers and senior leaders were approached to participate by email; carers were approached by telephone if they consented to be contacted for this purpose during initial trial recruitment.

METHODS

We used mixed methods including training observations, qualitative interviews, and online questionnaires at different time points to capture the perspectives of different stakeholders during the course of the study (Figure 1). Triangulating methods and incorporating multiple perspectives and experiences aimed to increase the credibility and trustworthiness of our findings [16].

Qualitative semi-structured interviews were conducted to provide rich descriptions of experiences and perspectives of both carers and staff. We conducted telephone interviews with carers as soon as possible after joining the main trial. Carer interviews were completed by members of the trial team (EP, KWN) and an experienced qualitative researcher (CM) between May 2017 and June 2018. Each carer was interviewed at the point of study consent and, if they agreed, again after primary outcomes had been collected at three months. Topic guides (supplementary material 1) were informed by the research aim and feedback from the RUG, and explored carers' experiences of support and their perceptions of the impact of support received.

Staff interviews were conducted with cluster frontline staff, managers and senior leaders between August and November 2018. All staff interviews were conducted by SD who had not been involved with staff training or support. Staff interviews were conducted face-to-face or over the telephone, depending on staff location and preference. Interview topic guides (supplementary material 2) were informed by NPT and aimed to capture context and carer support practices, and strategic organisational priorities (relevant for managers and senior leadership). For intervention-allocated frontline staff, additional questions explored changes to practice, mechanisms that supported changes, and perceived outcome of changes to practice. Researchers made field notes and reflections after all interviews, which lasted between 30-60 minutes. Interviews were audio recorded with anonymised transcripts, field notes, and reflections uploaded to Nvivo software to facilitate thematic analysis.

Online questionnaires were distributed via email to all frontline staff involved in OSCARSS at three separate time points: T1 pre-randomisation of clusters in August 2016, T2 and T3 post-randomisation in August 2017 and July 2018 respectively. Questionnaires asked about existing practice, attitudes towards supporting carers, the wider

For peer review only - http://bmjopen.bmj.com/site/about/guidelines.xhtml

context within which staff worked, and demographic information. At T2 and T3, the questionnaire for intervention arm staff additionally included items based on the NOrmalization MeAsure Development (NoMAD)[17] (supplementary material 3). NoMAD contains Likert items designed to explore intervention implementation, based on NPT.

Staff training in the intervention arm was typically delivered by the stroke organisation over half day group sessions and observed by KWN and EP. Field notes were taken to capture: perceived engagement of attendees with the training; whether all components of the training had been delivered as intended; types of questions asked by attendees and responses given. Staff also completed training feedback forms to explore their understanding of key components of the intervention.

Ethics approval was obtained from the North West Lancaster Research Ethics Committee (ref: 16/NW/0657) and University of Manchester (ref: AMBS-2016-22). All participants gave informed consent.

DATA ANALYSIS

Data were synthesised and thematically analysed using the four main constructs of NPT [15] which, as in previous studies, allowed us to combine qualitative and quantitative findings [18] into one explanatory framework [19]. Researchers experienced in NPT (SD and SK) led this method of data analysis as data were generated, following a continuous, iterative process [20]. We analysed at two levels: the individual and the organisation.

Data were initially coded inductively by several members of the research team (CM, EP, KWN, SD), with emerging findings coded deductively by process evaluation leads (SD and SK), guided by the NPT framework. This was an iterative process, where induction-deduction ensured codes fitted into a structure and related to each other in meaningful, study-important ways [19] and enabled prominent themes to be identified.

Questionnaire data provided summary information on usual practice of supporting carers within the stroke organisation and included NOMAD items relating to use and acceptability of the intervention. Other relevant data (e.g. training materials, observation field notes, and staff training feedback questionnaires) were reviewed throughout analysis (by SD and SK) to provide further context.

Draft findings were presented to multiple audiences, including internal PCPI collaborators (the RUG), Trial Management teams, and at external conferences and seminars. This enabled input from a multiplicity of viewpoints and supported reflective discussions to reach consensus on core, prominent themes.

RESULTS

Participants

Twenty-one carers of stroke survivors participated in first interviews (11 intervention, 10 control) and 11 agreed to follow up interviews (4 intervention, 7 control) (Table 2). Carers typically declined interviews when they felt they had nothing new to add.

Table 2 Carer characteristics

Key

SS needs score = independence level as reported by Carer (Min score = 1, Max score= 3)
C= control arm, I= intervention arm
M = Male, F = Female

For peer review only

Allocation	Gender	Age	Relation to Stroke Survivor	Stroke date	Ethnicity	SS needs score	Days from consent to interview	Status (1st/2nd interviews)
I	M	50-59	Partner	Feb 2017	White	2	33	completed 1&2
I	F	50-59	Daughter	May 2017	White	2.7	28	completed 1&2

For peer review only

I	M	60-69	Partner	May 2017	Asian	3	10	completed 1&2
I	F	60-69	Partner	Jan 2017	White	2.7	18	completed 1&2
I	F	30-39	Daughter	Jan 2017	Asian	1.7	4	opted out 2
I	F	50-59	Daughter	Aug 2017	White	3	21	opted out 2
I	F	50-59	Partner	June 2017	White	1	3	opted out 2
I	F	70-79	Partner	March 2017	White	1.7	19	opted out 2
I	M	70-79	Friend	March 2018	White	2	28	opted out 2
I	M	70-79	Partner	June 2017	White	1	29	opted out 2
I	F	70-79	Partner	Nov 2017	White other	1	65	opted out 2
C	F	40-49	Partner	Nov 2017	White	1	16	completed 1&2
C	F	60-69	Partner	Jan 2017	White	3	29	completed 1&2
C	M	60-69	Partner	Feb 2017	White	1.8	20	completed 1&2
C	M	60-69	Partner	Nov 2017	White	2.7	23	completed 1&2
C	F	60-69	Partner	Jan 2018	White	1.8	17	completed 1&2
C	F	70-79	Partner	Feb 2018	White	2.2	29	completed 1&2
C	F	70-79	Partner	March 2017	White	3	14	completed 1&2
C	F	60-69	Parent	July 2016	White	1.8	20	opted out 2
C	F	50-59	Daughter	Sep 2017	White	2.7	77	opted out 2
C	F	50-59	Daughter	Dec 2017	White	1.6	46	opted out 2

Thirty-one staff members were interviewed (frontline staff: 12 intervention, 8 control; managers and senior leaders across clusters: 11). Thirteen additional frontline staff declined interviews. Staff participants were based across England and Northern Ireland, included both full and part-time workers and length of service ranged from less than a year to 23 years.

We observed 25 hours of staff training on the CSNAT-Stroke intervention over six group sessions, including 47 intervention-allocated staff. Response rates to the online staff questionnaire were: T1 72% (62 pre-intervention), T2 48% (20 intervention, 21 control), T3 35% (20 intervention, 17 control). Due to staff turnover, the same staff did not necessarily answer each questionnaire. Questionnaire respondents were predominantly female, aged 43 and over, worked part-time, and length of service ranged from less than one year to over ten.

Themes

In this section, we draw upon themes identified through the NPT framework to report factors that impacted on implementation of the intervention (Table 3). For this purpose we mainly present data from the staff intervention arm (interviews and questionnaires T2 and T3), as well as from carers, managers and senior leaders.

Table 3 Themes identified through the NPT framework

NPT construct and definition	Relevant questions considered to guide analysis	Theme from data

For peer review only

**Coherence:** Understanding and valuing of the intervention	Was the intervention understood as different from usual practice?<br>What were the beliefs and behaviours that defined the new approach?<br>Was there a shared understanding of the intervention?<br>Was there a benefit identified in following the intervention?	**Limited understanding of different components of the intervention:** Although all staff valued the ethos of a carer-led approach to supporting carers, despite the training staff demonstrated a limited understanding of the whole intervention.
**Cognitive Participation:** Engagement in and activation of the intervention by individuals and groups	How was the intervention initiated?<br>Did staff and carers feel like legitimate participants in the intervention?<br>How were staff and carers enrolled and engaged in the intervention?<br>What promoted or inhibited participation in the intervention?	**Limited participation in the intervention:** Staff engaged with the intervention but struggled to persuade carers to participate and carers did not necessarily see themselves as legitimate participants.
**Collective Action:** How participants work to carry out the intervention	How was the intervention carried out?<br>What were the beliefs and behaviours of staff and carers that define and organise the work?<br>How did staff fit the new approach into their existing practice and context?<br>Who was best placed to make use of the intervention?	**The intervention was not fully implemented:** Key components of the approach (such as a separate conversation between carer and staff member, and completion of the action plan) were not always implemented.
**Reflexive Monitoring:** How the intervention is appraised and evaluated	What factors promoted or inhibited appraisal of the intervention?<br>How was the intervention monitored and evaluated?<br>How did staff and carers appraise the practice?<br>Was the intervention modified or adapted?	**Limited opportunities to share learning and ongoing support for the intervention:** All staff positively appraised the intervention as they understood it, but there were limited opportunities to share learning and receive ongoing support from across the stroke organisation.

We present our findings by focusing at the individual (staff and carer) and then at the organisational level. Taking both levels into account helps identify the individual behavioural changes and agency, as well as the organisational structural and cultural changes required to implement a complex intervention [14].

Individual staff and carer level factors

Carers in both arms of the study reported doing a wide range of caring tasks including personal care, practical tasks, and financial administration. They described the impact of caring on their emotional and physical wellbeing, restricted social life, having to give up work, and financial implications:

> "I'm 77 and it is hard work… it doesn't switch off." [Carer 1, intervention, interview 1]

However, during support visits carers did not always feel able to consider their own needs as their priority was the person they were caring for. This inhibited their **Cognitive Participation** in the intervention. Frontline staff saw this as a persistent issue, despite training for the intervention that included how to introduce CSNAT-Stroke during initial contact in a way that was sensitive to carer self-identification:

> "Where there's sometimes debate is when you're labelling someone as a carer and often someone's wife, for example, would say - I'm not his carer, I'm his wife." [Frontline staff member 1, intervention, interview]

Carers in the control arm appraised the support they received from the stroke organisation just as positively as carers did in the intervention arm, particularly when they received little support from other organisations. Generally all carers interviewed said that they were grateful to have somebody interested in their needs and able to provide practical help.

More than half of intervention arm staff respondents to T2 and T3 questionnaires said they could see how the intervention differed from their usual way of working and could be easily integrated into current practice. However, in the interviews where this issue was explored in detail there were mixed perceptions of the difference, particularly for staff who had been in the role for a number of years:

For peer review only

> "I'd say it was quite similar, it's just the way it's outlaid isn't it really? What I do is not on paper as such… I've got experience behind me and I've worked with carers for a long time." [Frontline staff member 2, intervention, interview]

The desire for a structured, formalised approach to offering carer support came through strongly from all staff, including senior leaders. Over half the intervention respondents to T2 and T3 agreed that staff in their service had a shared understanding of the intervention. However, the intervention was primarily perceived as solely the paper-based assessment needs tool:

> "It's been a real pleasure to use, I consider it useful …in the beginning it was like okay great, more paper, but actually it's one of my most useful pieces of paper and I've got a lot of paper." [Frontline staff member 3, intervention, interview]

The lack of **Coherence** of the intervention is reflected in descriptions by staff of not fully implementing it and the similar accounts of support received by carers in both trial arms. Staff also appeared to see certain parts of the intervention, such as the action plan, as duplicating existing practice rather than a route to empowering carers to take ownership of actions for themselves:

> "I have to confess, I didn't [use the action plan]… I would follow it up in a letter … I don't see the point in doing [the action plan] …I'd be duplicating it." [Frontline staff member 4, intervention, interview]

Frontline staff positively appraised the OSCARSS training and, in feedback forms, highly endorsed that they understood each stage of the intervention and felt confident to deliver it. Despite this, interviews suggested that the training and ongoing support had not been sufficient to equip staff with a full understanding of the carer-led process of the intervention. In particular, the initiation of a separate conversation with a carer to discuss their needs is an important part of the intervention that was championed by the RUG. Staff described that historically these separate conversations tended to be intermittent and initiated by carers (often on the doorstep at the end of a visit), whereas OSCARSS training encouraged staff to routinely initiate a separate conversation and normalise this in advance of a visit. Whilst staff saw the value of this in principle, they did not always initiate it in practice and instead many reported asking carers for the first time about meeting separately during the visit and in front of the stroke survivor:

> "What I find is I'll introduce it but people don't want to go off and talk on their own. They want to stay with the stroke survivor. Some people do, but some people don't. They said, oh no, that's fine, we can talk quite openly with each other, so you can't make people go and talk on their own." [Frontline staff member 5, intervention. interview]

The lack of full implementation was also evident in the needs assessment process. Staff would often assume what carers' needs might be and anticipate not being able to meet them, which led to a reluctance to comprehensively discuss needs:

> "To sort of say, okay, I recognise you're struggling with that and I don't have a solution… you're perceiving them to think, what was the point of asking me if I needed help - you've just highlighted the fact I need help and there's no services to help me." [Frontline staff member 1, intervention, interview]

Frontline staff had a well-meaning, strong desire to practically meet carer needs and 'fix' problems. However, core tenets of the carer-led approach of the intervention were that needs should be heard to normalise needs and permit collaborative problem-solving, tailoring support to the carer's priorities.

Despite the lack of full implementation, components of the intervention that were understood were positively appraised by individual staff. For example, many expressed a desire to continue using the needs assessment tool in their meetings with carers beyond the trial. Managers were also keen to continue a systematic, structured approach to supporting carers.

The organisational level and contextual factors

The focus on carers and the carer-led approach of the intervention aligned with organisational values and was strongly supported by senior leaders and managers. However, managers did not have a comprehensive understanding of the intervention and whilst frontline staff perception of management support for the intervention increased over time, they saw training for and communications about the intervention as coming from the research team rather than the organisation:

For peer review only

> “I don’t think really the [organisation] got involved much ’cause it was separate, wasn’t it? Some [services] were included, some weren’t, so I think they just left [the research team] to it and let you support us.” [Frontline staff member 2, intervention, interview]

Frontline staff were viewed as most appropriate to deliver the intervention through **Collective Action**, although over half of T2 and T3 respondents in the intervention arm were neutral on whether work was assigned to those with skills appropriate to the intervention. At T2 and T3 frontline staff expressed openness to working with colleagues in new ways to use the intervention and in interviews frontline staff expressed a desire to learn from or share experiences with other teams.

However, there appeared to be few opportunities for shared learning on the intervention. Staff usually worked from home and did not have regular contact with each other, so opportunities were unlikely to happen without managerial planning. **Reflexive Monitoring** was limited with no systematic monitoring or evaluation of the intervention within the organisation, although almost all of T2 and T3 intervention respondents agreed that feedback could be used to improve the intervention.

During OSCARSS, services across the organisation experienced significant reorganisation internal staffing changes and commissioning changes. In interviews some frontline staff described difficulty participating in the intervention within the context of internal and external changes and pressures:

> “OSCARSS hasn’t been a priority at all, we’ve been going through a lot of changes, we’ve lost some of our funding, we’ve had commissioned services being cut. There has been much more priority than doing the trial.” [Frontline staff member 6, intervention, interview]

Just over half T2 and T3 intervention respondents agreed or strongly agreed that sufficient resources were available to support the intervention. However, in staff interviews a lack of time for and during visits to stroke survivors and carers was highlighted as inhibiting participation in the intervention:

> “I think time would be the main thing, because… a lot of [frontline staff] just wouldn’t necessarily factor the time in to a visit because they’re already quite long, and there is already quite a lot of things to do within that visit.” [Senior Leader, interview]

DISCUSSION

The OSCARSS process evaluation helps us understand the context in which the intervention was delivered within the trial and provides some explanation of why no meaningful added benefit was evident when comparing intervention with usual practice. We identified significant challenges to successful implementation of the intervention at both individual and organisational levels. Frontline staff were mainly positive about the intervention, insofar as they wished to enhance carer support, but they did not have a full understanding of the *carer-led* aspects of the intervention. The training that frontline staff received through OSCARSS (half a day face-to-face or 1-3 hours over Skype) and ongoing informal support from the research team was not sufficient to provide this understanding. Frontline staff cared deeply about supporting carers, but their tendency to anticipate and ‘fix’ needs limited opportunities for the approach to be carer-led. Key stages of the intervention were also not routinely implemented, such as a separate conversation and completion of an action plan. Consistent with this finding, interviews with carers demonstrated little difference in support provided across the trial’s intervention and control arms. Despite positive engagement and organisational enthusiasm for OSCARSS, the intervention was not delivered as intended. The findings demonstrate challenges to delivery of carer-led assessment and support, including overcoming the reluctance of carers to express their own needs, frontline staff anticipating carer needs, and a need for ongoing organisational support and learning.

Although there is strong evidence for the need to support carers of stroke survivors [21, 22] and a statutory requirement to do so [6] those considering implementing carer-led interventions should be aware of the challenges and solutions highlighted by OSCARSS. Carers in our study described the range of tasks they were responsible for in providing care and the impact this has on their lives. However, in line with other studies the carers did not always feel ready to accept support for themselves and did not necessarily see themselves as “carers” [23, 24] and the intervention was not implemented in a way to address this issue. Using NPT emphasised that motivation and ‘buy in’ for an intervention relies on engagement of both practitioner and service recipient to ‘enroll’ themselves as eligible and appropriate for an intervention to work. This may be particularly challenging for carers. Our findings contribute to this often overlooked yet vital aspect in implementation theory [25] and our multi-stakeholder analysis enabled us to explore both individual and wider contextual factors that impact on implementation in practice.

For peer review only - http://bmjopen.bmj.com/site/about/guidelines.xhtml

Our process evaluation has shown the difficulty implementing a complex intervention that follows a carer-led ethos, despite strong organisational enthusiasm and staff willingness to support carers. Our findings show that staff would have welcomed additional ongoing support and shared learning opportunities to gain a full understanding of the carer-led approach. Support mechanisms used in previous CSNAT intervention implementations, such as developing staff champions [26, 27] may have ensured that managerial commitment was communicated to frontline staff. Many carers across both trial arms said that frontline staff in the organisation were one of their main sources of support and this was highly valued. In OSCARSS, the intervention was developed to be used within the pragmatic organisational context of individual staff supporting both stroke survivor and carer. However, our findings highlight the barriers for coordinators to fully support both individuals within their available resources and limited time. Our findings also highlight the particular complexity of meeting needs of carers who struggle to recognise their own needs, and the need to consider timing of support and how to sensitively and appropriately facilitate conversations with carers and stroke survivors.

The process evaluation was led by experienced researchers separate to the trial team and used multiple methods over time to generate rich and in-depth data. It used NPT to bring together these data that provided a range of perspectives at different time points and sampled from both trial arms across clusters. Through examining implementation at the individual and organisational level, the process evaluation offers insight into why the intervention was not fully implemented and contributes to knowledge on implementing carer-led approaches in health care. A limitation to the study was that observation of the intervention in real-time was not conducted as it was not practical within this large-scale study of 35 clusters. However, we recruited carers and staff from different geographical areas within the trial and triangulated multiple data to get an understanding of implementation from the perspective of several stakeholder groups.

The process evaluation showed that the intervention was not fully implemented as intended, therefore we cannot say whether it may provide a benefit to carers of stroke survivors. Using NPT we have highlighted areas that need to be addressed in future research of this intervention or similar person-led approaches to supporting carers of stroke survivors. Considerations include firstly, how to train and support staff, including strategies to help with engaging carers reluctant to prioritise their own need. Secondly, ensuring clear communication pathways to ensure adoption of the intervention, involving management goals top down to frontline staff and also bottom up enabling practitioners to raise implementation difficulties and have them resolved. Thirdly, having a network of staff champions as role models and for problem solving. Fourthly, clarity at the outset about resources needed for implementation and sustainability.

CONCLUSIONS

This paper shows the value of process evaluation in helping to interpret neutral trial results, such as OSCARSS. It demonstrates how both staff and service user perceptions and organisational demands can impede the delivery of key intervention components, even with significant buy-in to the need for the intervention itself. Our findings identified certain challenges, as well as opportunities, that contribute to knowledge on implementing carer-led support interventions. We recommend that future intervention work should concentrate on addressing organisational factors which can both help and hinder implementation and that all levels within the organisation are engaged in implementation, including undertaking training to have a full understanding of all components of the intervention.

AUTHOR CONTRIBUTIONS

All authors (SD, SK, KWN, CM, GG, GE, SR, AB, EP) contributed to the design of this study. SD, KWN, CM and EP contributed to recruitment, collected the data and contributed to the analysis. All authors (SD, SK, KWN, CM, GG, GE, SR, AB, EP) contributed to interpretation of findings and writing of the manuscript. Jessica Stolberg contributed to collection of questionnaire data and Verity Longley contributed to analysis. The trial steering committee contributed to the study design and interpretation of findings. The study specific carer advisory research group contributed to the study design and interpretation of findings: Kelly Burke, Christine Halford, Natalie Halford, Geoff Heathcote, Kath Purcell and Ben Wright.

ACKNOWLEDGEMENTS

The authors would like to thank all of the carers and staff members who participated and contributed to this study. We would like to thank members of the supportive NIHR CLARHC GM team who helped make the study possible including: Caroline O'Donnell (data analyst); Alison Littlewood and Katy Rothwell (programme managers); Sam Wilkinson, Amy Woodhouse and Rose Crees (administrative assistants); Aneela Macavoy and

For peer review only

Zoe Ashton (research facilitators). We also wish to thank and acknowledge Stroke Association staff, including Chris Larkin, Elaine Roberts, Cass Saunders and Kathryn Staley.

REFERENCES

1 Crichton SL, Bray BD, McKevitt C, et al. Patient outcomes up to 15 years after stroke: survival, disability, quality of life, cognition and mental health. *J Neurol Neurosurg Psychiatry Res*, 2016;87:1091–1098.

2 Noonan MC, Wingham J, Taylor RS. 'Who Cares?' The experiences of caregivers of adults living with heart failure, chronic obstructive pulmonary disease and coronary artery disease: a mixed methods systematic review *BMJ Open,* 2018;8:e020927.

3 Quinn K, Murray C, Malone C. Spousal experiences of coping with and adapting to caregiving for a partner who has a stroke: a meta-synthesis of qualitative research. *J Disabil Rehabil,* 2014;36:185–198.

4 Carers UK. NHS England's Long Term Plan and what it means for carers, 2019. Available: https://www.carersuk.org/for-professionals/policy/policy-library/the-nhs-long-term-plan-and-what-it-means-for-carers [accessed 12 August 2019]

5 Department of Health & Social Care. Carers Action Plan 2018 to 2020: Supporting carers today. Available www.nationalarchives.gov.uk/doc/open-government-licence/ [accessed 6 September 2019]

6 Department of Health, Care Act. 2014. Available: http://www.legislation.gov.uk/ukpga/2014/23/contents/enacted/data.htm [accessed 21 November 2019]

7 Ewing G, Grande GA. Development of a Carer Support Needs Assessment Tool (CSNAT) for end-of-life care practice at home: *Palliat Med,* 2012;27:244-256.

8 Ewing G, Austin L, Grande G. The role of the Carer Support Needs Assessment Tool in palliative home care: A qualitative study of practitioners' perspectives of its impact and mechanisms of action. *Palliat Med*, 2016;30:392–400.

9 Ewing G, Brundle C, Payne S. The Carer Support Needs Assessment Tool (CSNAT) for Use in Palliative and End-of-life Care at Home: A Validation Study. *J Pain Symptom Manage*, 2013;46:395-405.

10 Aoun SM, Grande G, Howting D, et al. The impact of the carer support needs assessment tool (CSNAT) in community palliative care using a stepped wedge cluster trial, *PLoS One*, 2015;10:4.

11. Patchwood E, Rothwell K, Rhodes S, et al. Organising Support for Carers of Stroke Survivors (OSCARSS): study protocol for a cluster randomised controlled trial, including health economic analysis. *Trials*, 2019;20:19.

12 Grant A, Treweek S, Dreischulte T, et al. Process evaluations for cluster-randomised trials of complex interventions: a proposed framework for design and reporting. *Trials*, 2013;14:15.

13 Moore GF, Audrey S, Barker M, et al. Process evaluation of complex interventions: Medical Research Council guidance. *BMJ*, 2015;350:h1258.

14 May CR, Cummings A, Girling M, et al. Using Normalization Process Theory in feasibility studies and process evaluations of complex healthcare interventions: a systematic review. *Implement Sci*, 2018;13:80.

15 May C, Finch T. Implementing, Embedding, and Integrating Practices: An Outline of Normalization Process Theory. *Sociology*, 2009;43:535–554.

16 Patton M. Qualitative evaluation and research methods. Beverly Hills, CA: Sage 1990.

17 May CR, Finch T, Ballini L, et al. Evaluating complex interventions and health technologies using normalization process theory: development of a simplified approach and web-enabled toolkit. *BMC Health Serv Res*, 2011;11:245

18 Lewis NV, Dowrick A, Sohal A, et al. Implementation of the Identification and Referral to Improve Safety programme for patients with experience of domestic violence and abuse: A theory-based mixed-method process evaluation. *Health Soc Care Community*, 2019;27:e298-e312.

19 Moran-Ellis J, Alexander VD, Cronin A, et al. Triangulation and integration: Processes, claims and implications. *Qual Res*, 2006;6:45–59.

For peer review only

20 Miles MB, Huberman AM. Qualitative Data Analysis: An expanded sourcebook. Thousand Oaks, CA: Sage 1994.

21 Haley WE, Roth DL, Hovater M, et al. Long-term impact of stroke on family caregiver well-being: a population-based case-control study. *Neurology*, 2015;84:1323–1329.

22 Kruithof WJ, Post MWM, Van Mierlo ML, et al. Caregiver burden and emotional problems in partners of stroke patients at two months and one year post-stroke: Determinants and prediction. *Patient Educ Couns*, 2016;99: 1632–1640.

23 Knowles S, Combs R, Kirk S, et al. Hidden caring, hidden carers? Exploring the experience of carers for people with long-term conditions. *Health Soc Care Community*, 2016;24:203–213.

24 Cameron JI, Gignac MAM. "Timing It Right": A conceptual framework for addressing the support needs of family caregivers to stroke survivors from the hospital to the home. *Patient Educ Couns*, 2008;70: 305–314.

25 Kennedy A, Rogers A, Chew-Graham C, et al. Implementation of a self-management support approach (WISE) across a health system: a process evaluation explaining what did and did not work for organisations, clinicians and patients. *Implement Sci*, 2014;9:129.

26 Diffin J, Ewing G, Harvey G, et al. Facilitating successful implementation of a person-centred intervention to support family carers within palliative care: a qualitative study of the Carer Support Needs Assessment Tool (CSNAT) intervention. *BMC Palliat Care*, 2018;17:129.

27 Austin L, Ewing G, Grande, G. Factors influencing practitioner adoption of carer-led assessment in palliative homecare: A qualitative study of the use of the Carer Support Needs Assessment Tool (CSNAT), *PLoS One*, 2017;12:6.

28 Hoffman TC, Glasziou PP, Boutron I, et al. Better reporting of interventions: template for intervention description and replication (TIDieR) checklist and guide, *BMJ*, 2014;348:g1687

For peer review only

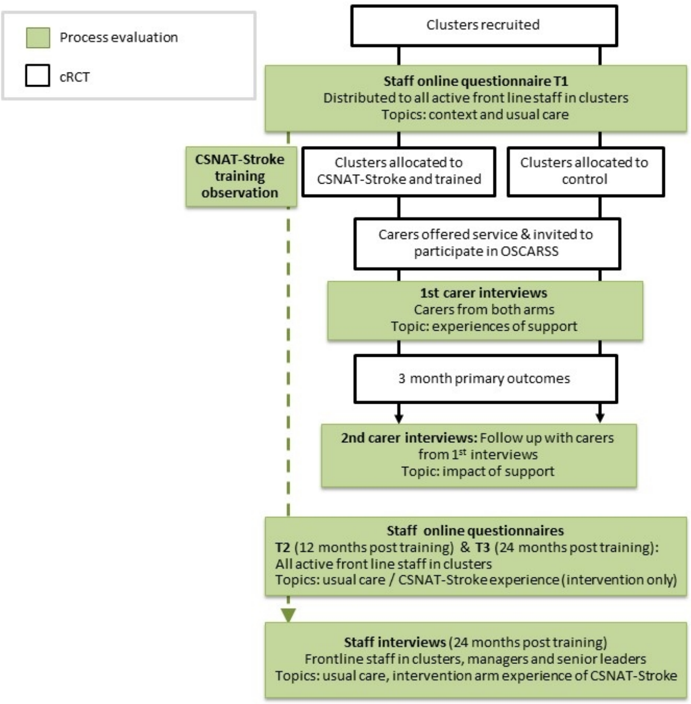

Figure 1 Process evaluation methods overview within the OSCARSS trial

254x190mm (96 x 96 DPI)

SUPPLEMENTARY MATERIAL 1

Topic guides for carer interviews

First interviews: Questions about experiences of support

I'd like to speak to you about contact you've had from [stroke organisation].

- Can you talk me through what happened during the visit?
    - Did you talk with X on your own or with others? (how was that / how did that go?)
    - Was any paperwork used? Possible additional probs : Can you tell me more about the paperwork you used? How was that / how did that go?
    - Did you keep a copy of any paperwork? If so, do you ever refer to it / find it helpful?
    - Did you talk about any support that you might need , as someone who is providing care/ support to <INSERT RELATIONSHIP eg mum >> How was that / How did that go?
    - Did you feel that you or the staff member 'took the lead' when talking? (Could re-visit 'paperwork' prompts here too)
- Did you come up with ideas of how to address the needs/issues you talked about? Can you talk me through them ?
    - Depending on if/how addressed earlier could repeat: Did you feel that you or the coordinator 'took the lead' when coming up with ideas?
    - Were you happy with the solutions/ ideas for support?
    - Were there any things that are not being addressed/ that it wasn't possible to address? What were these? How do you feel about this?
- Do you have any plans to talk with (name) / the stroke organisation again about support for you?
- What kind of support did you expect when you accepted a [stroke organisation] visit?
- Did it meet your expectations?
- Overall, what were your impressions of the visit?
- Are there any practical ways that the [stroke organisation] has influenced your own life as a caregiver? [Or have you found your own means of support, without reference to the stroke organisation?]

Second interviews: Questions about perceptions of impact support

[Last time we spoke] You told me about the things that were suggested that might be of help. Did you try any of these things?

- If no, why not?

If yes :

- Did they help? Have you continued to use this support?
    - Why/why not?
    - Has it lead to other things that have helped you as a carer?
- Is there any support you would have liked but felt like you didn't get?
    - Why? / how do you think it would have helped?
    - Has that changed over time?

For peer review only

- Overall - How did you find the [stroke organisation] in terms of supporting you as a carer? [Was it helpful/unhelpful?] Explore more:
  - Did it make any difference to you as a carer? If yes - in what way?
  - Has the support you received made any difference to the person you care for?
  - Is there anything you would have liked to have been done differently?

Finally

- Is there anything else you would like to tell us about the support you received / support for carers that we haven't covered?

For peer review only

SUPPLEMENTARY MATERIAL 2

Topic guides for staff

Frontline staff topic guide

Current practice

- Could you tell me briefly about your role [core tasks/expectations] within the stroke organisation? (how long?)
    - Could you tell me about your role in relation to supporting carers?
- Could you talk me through your current process of supporting carers?
    - Identifying (and defining) carers
    - Initial contact (face to face/ by phone; planned or unplanned? Always with stroke survivor or alone – and how does this happen?)
    - Identifying needs (paperwork; recording information? Action plan / follow up?)
    - Are there any other things that help you support carers? (e.g. local services)
    - Anything that prevents you from supporting carers?
- How have you found the OSCARSS recruitment process?

Changes to current practice

- Apart from OSCARSS are there any other factors that have impacted on how you support carers?
    - Has the CSNAT-Stroke been discussed within or/and across services? (e.g. through networking/learning events) – **control arm, skip to final question**

Intervention arm only:

- Have you been using CSNAT-Stroke during your visits that have involved carers? (If no – reasons why not? If yes – how did you use it? How have you found using it?)
- How would you describe CSNAT-Stroke?
- Similarities or differences of CSNAT-Stroke to how you've worked with carers before? If no difference – why is this?
- Any occasions where you would use an alternative process to address carer needs?
    - Any adaptation of CSNAT-Stroke or deliberately doing something else? Why?

Mechanisms that have supported changes

- Can you tell me about anything that has supported your use and understanding of CSNAT-Stroke
- Has there been anything that has hindered your use of CSNAT-Stroke? What could have helped overcome these hindrances?

Outcomes

- Through using CSNAT-Stroke do you feel like this has had any impact on your work with carers? Any impact on your work with stroke survivors?
- Do you think you would you continue to follow CSNAT-Stroke in your work with carers (why/why not)? What would you need to support the continued use of CSNAT-Stroke (on an individual and organisational level)?
- What is your overall opinion of CSNAT-Stroke?
- Is there anything else you would like to tell us about how you support carers that we haven't covered?

For peer review only

Managers and Senior Leaders: Topic guide

Current practice

- Could you tell me about your role within the organisation? (Involvement in supporting carers/ knowledge of how carers are supported?)
- How important do you feel supporting carers is within the organisation?
- What is needed to provide carer support? Who in the organisation should provide carer support?

Changes to current practice

- How much do you know about CSNAT-Stroke? (similarities or differences to standard practice etc.)
- How did you find out about CSNAT-Stroke?

Mechanisms that have supported changes

- Do you know of anything that has helped or hindered the use of CSNAT-Stroke?
- What structures would need to be in place to support the use of an approach like CSNAT-Stroke?

Outcomes

- Has the use of CSNAT-Stroke within the organisation created any changes?
    - Anything else that might have contributed to these changes?
- Do you think that the use of CSNAT-Stroke will be continued to support carers (why/why not)?
    - Does it feel like part of the organisation’s approach to supporting carers now?
    - What would the organisation need to support the continued use of CSNAT-Stroke?
    - Who would be best placed/resourced to provide the training for CSNAT-Stroke?
- What is your overall opinion of CSNAT-Stroke?
- Is there anything else you would like to tell us about how carers are supported or CSNAT-Stroke that we haven’t covered?

For peer review only

SUPPLEMENTARY MATERIAL 3

T2 and T3 questionnaire items based on NoMAD for frontline staff in the intervention arm only

	T2 questionnaire (N=20)			T3 questionnaire (N=20)		
**Item**	**Agree *n***	**Neutral *n***	**Disagree *n***	**Agree *n***	**Neutral *n***	**Disagree *n***
**Coherence**						
I can see how the CSNAT Stroke differs from usual ways of working	14	4	2	11	8	1
Staff in this service have a shared understanding of the purpose of the CSNAT-Stroke	13	7	0	10	7	3
I can see the potential value of the CSNAT-Stroke for my work	17	0	3	18	2	0
I understand how the CSNAT-Stroke affects the nature of my own work	18	2	0	17	2	1
**Cognitive participation**						
I believe that participating in the CSNAT-Stroke is a legitimate part of my role	15	3	2	16	4	0
There are key people in the Stroke Association who drive the CSNAT Stroke forward and get others involved	5	11	4	5	12	3
I'm open to working with colleagues in new ways to use the CSNAT-Stroke	16	2	2	16	4	0
**Collective action**						
I can easily integrate the CSNAT-Stroke into my working practice.	14	1	5	13	6	1
Sufficient resources are available to support the CSNAT Stroke	11	3	6	13	3	4
Sufficient training is provided to enable staff in this service to implement the CSNAT-Stroke	14	2	4	17	3	0
I have confidence in other people's ability to use the CSNAT-Stroke	10	10	0	11	9	0
Work is assigned to those with skills appropriate to the CSNAT-Stroke	5	13	2	7	12	1
Management adequately supports the CSNAT-Stroke	1	13	6	10	7	3
**Reflexive monitoring**						
Staff in this service agree that the CSNAT-Stroke is worthwhile	13	5	2	13	7	0
I value the effects that the CSNAT-Stroke has had on my work	12	5	3	15	5	0
Feedback about the CSNAT-Stroke can be used to improve it in the future	16	4	0	20	0	0

For peer review only

I can modify how I work with the CSNAT-Stroke	16	1	3	13	6	1

For peer review only

CHALLENGES IMPLEMENTING A CARER SUPPORT INTERVENTION WITHIN A NATIONAL STROKE ORGANISATION: FINDINGS FROM THE PROCESS EVALUATION OF THE OSCARSS TRIAL

Consolidated criteria for reporting qualitative studies (COREQ): 32-item checklist

No	Item	Guide questions/description	Our response
**Domain 1: Research team and reflexivity**			
**Personal Characteristics**			
1	Interviewer/facilitator	Which authors conducted the interviews	SD conducted all the staff interviews, KWN, EP and CM conducted the carer interviews.
2	Credentials	What were the researcher's credentials? E.g. PhD, MD	SD, EP and CM have PhDs. KWN has an MSc.
3	Occupation	What was their occupation at the time of the study?	SD was Research Associate. EP and CM were Research Fellows. KWN was Research Project Manager.
4	Gender	Was the researcher male or female?	Researchers were all female
5	Experience and training	What experience or training did the researcher have?	All researchers had experience and training in qualitative research methods
**Relationship with participants**			
6	Relationship established	Was a relationship established prior to study commencement?	SD had no relationship established with staff interviewed prior to study commencement. KWN, EP and CM had no relationship established with carers interviewed prior to study commencement.
7	Participant knowledge of the interviewer	What did the participants know about the researcher? e.g. personal goals, reasons for doing the research	All participants were given Participant Information Sheets which explained the

For peer review only

			research and the researchers' role in the study.
8	Interviewer characteristics	What characteristics were reported about the interviewer/facilitator? e.g. Bias, assumptions, reasons and interests in the research topic	There are no relevant characteristics to report about the interviewers.
**Domain 2: study design**			
**Theoretical framework**			
9	Methodological orientation and Theory	What methodological orientation was stated to underpin the study? e.g. grounded theory, discourse analysis, ethnography, phenomenology, content analysis	Process evaluation informed by Normalisation Process Theory (NPT).
**Participant selection**			
10	Sampling	How were participants selected?	Purposive sample
11	Method of approach	How were participants approached? e.g. face-to-face, telephone, mail, email	Staff were approached by email, carers were approached by telephone if they gave consent when recruited to the trial.
12	Sample size	How many participants were in the study?	21 carers and 31 staff were interviewed. We received 140 responses to a staff questionnaire at 3 different time points.
13	Non-participation	How many people refused to participate or dropped out? Reasons?	13 staff members chose not to be interviewed for reasons including they did not have time or felt they had nothing to say in an interview. 11 carers did not participate in second interviews, mainly because they felt they had nothing more to add since their first interview.
**Setting**			

For peer review only

14	Setting of data collection	Where was the data collected? e.g. home, clinic, workplace	Over the telephone for all carers and most staff, and in the workplace for some staff.
15	Presence of non-participants	Was anyone else present besides the participants and researchers?	No
16	Description of sample	What are the important characteristics of the sample? e.g. demographic data, date	Carers and staff members in the stroke organisation who support carers as part of their role.
**Data collection**			
17	Interview guide	Were questions, prompts, guides provided by the authors? Was it pilot tested?	Interviews were semi-structured (questions and prompts included in supplementary material). No pilot testing took place.
18	Repeat interviews	Were repeat interviews carried out? If yes, how many?	Repeat interviews were carried out with 11 carers.
19	Audio/visual recording	Did the research use audio or visual recording to collect the data?	All interviews were audio recorded
20	Field notes	Were field notes made during and/or after the interview or focus group?	Field notes were made after each interview
21	Duration	What was the duration of the interviews or focus group?	30-60 minutes
22	Data saturation	Was data saturation discussed?	Yes by the researchers involved in analysis on an ongoing basis
23	Transcripts returned	Were transcripts returned to participants for comment and/or correction?	No, but the content of interviews were summarised at the end of each interview and checked with participants.
**Domain 3: analysis and findings**			
**Data analysis**			
24	Number of data coders	How many data coders coded the data?	5
25	Description of the coding tree	Did authors provide a description of the coding tree?	Yes this was based on the NPT framework

For peer review only

26	Derivation of themes	Were themes identified in advance or derived from the data?	Analysis was both deductive using NPT and deductive drawing on the data
27	Software	What software, if applicable, was used to manage the data?	Nvivo
28	Participant checking	Did participants provide feedback on the findings?	Some staff participants gave feedback on the findings. Our Research User Group of carers also provided feedback.
29	Quotations presented	Were participant quotations presented to illustrate the themes / findings? Was each quotation identified? e.g. participant number	Participant quotations are presented and identified.
30	Data and findings consistent	Was there consistency between the data presented and the findings?	Yes
31	Clarity of major themes	Were major themes clearly presented in the findings?	Yes
32	Clarity of minor themes	Is there a description of diverse cases or discussion of minor themes?	We were restricted by the word count so have focused on major themes in this paper.

For peer review only

BMJ Open

CHALLENGES IMPLEMENTING A CARER SUPPORT INTERVENTION WITHIN A NATIONAL STROKE ORGANISATION: FINDINGS FROM THE PROCESS EVALUATION OF THE OSCARSS TRIAL

Journal:	*BMJ Open*
Manuscript ID	bmjopen-2020-038129.R1
Article Type:	Original research
Date Submitted by the Author:	07-Jul-2020
Complete List of Authors:	Darley, Sarah; The University of Manchester, Alliance Manchester Business School<br>Knowles, Sarah; The University of Manchester, Alliance Manchester Business School<br>Woodward-Nutt, Kate; Salford Royal Hospitals NHS Trust<br>Mitchell, Claire; The University of Manchester, Manchester Academic Health Sciences Centre (MAHSC), Division of Neuroscience and Experimental Psychology<br>Grande, Gunn; The University of Manchester, Manchester Academic Health Sciences Centre (MAHSC), Division of Nursing, Midwifery and Social Work<br>Ewing, Gail; University of Cambridge, Centre for Family Research<br>Rhodes, Sarah; The University of Manchester, Manchester Academic Health Sciences Centre (MAHSC), Centre for Biostatistics, Division of Population Health Services Research and Primary Care, School of Health Sciences:<br>Bowen, Audrey; The University of Manchester, Manchester Academic Health Sciences Centre (MAHSC), Division of Neuroscience and Experimental Psychology<br>Patchwood, Emma; The University of Manchester, Manchester Academic Health Sciences Centre (MAHSC), Division of Neuroscience and Experimental Psychology
**Primary Subject Heading**:	Qualitative research
Secondary Subject Heading:	Public health, Health services research
Keywords:	Stroke < NEUROLOGY, QUALITATIVE RESEARCH, PUBLIC HEALTH

SCHOLARONE™ Manuscripts

I, the Submitting Author has the right to grant and does grant on behalf of all authors of the Work (as defined in the below author licence), an exclusive licence and/or a non-exclusive licence for contributions from authors who are: i) UK Crown employees; ii) where BMJ has agreed a CC-BY licence shall apply, and/or iii) in accordance with the terms applicable for US Federal Government officers or employees acting as part of their official duties; on a worldwide, perpetual, irrevocable, royalty-free basis to BMJ Publishing Group Ltd ("BMJ") its licensees and where the relevant Journal is co-owned by BMJ to the co-owners of the Journal, to publish the Work in this journal and any other BMJ products and to exploit all rights, as set out in our licence.

The Submitting Author accepts and understands that any supply made under these terms is made by BMJ to the Submitting Author unless you are acting as an employee on behalf of your employer or a postgraduate student of an affiliated institution which is paying any applicable article publishing charge ("APC") for Open Access articles. Where the Submitting Author wishes to make the Work available on an Open Access basis (and intends to pay the relevant APC), the terms of reuse of such Open Access shall be governed by a Creative Commons licence – details of these licences and which Creative Commons licence will apply to this Work are set out in our licence referred to above.

Other than as permitted in any relevant BMJ Author's Self Archiving Policies, I confirm this Work has not been accepted for publication elsewhere, is not being considered for publication elsewhere and does not duplicate material already published. I confirm all authors consent to publication of this Work and authorise the granting of this licence.

For peer review only

TITLE PAGE

CHALLENGES IMPLEMENTING A CARER SUPPORT INTERVENTION WITHIN A NATIONAL STROKE ORGANISATION: FINDINGS FROM THE PROCESS EVALUATION OF THE OSCARSS TRIAL

Corresponding author
Name: Dr Sarah Darley
Address: Suite 1 Williamson Building, Centre for Primary Care and Health Services Research, Faculty of Biology, Medicine and Health, University of Manchester, Oxford Road, Manchester M13 9QQ, UK
Email: Sarah.Darley@manchester.ac.uk
Telephone: +44 161 275 7687

Authors: Sarah Darley, Sarah Knowles, Kate Woodward-Nutt, Claire Mitchell, Gunn Grande, Gail Ewing, Sarah Rhodes, Audrey Bowen, and Emma Patchwood on behalf of the OSCARSS Research Team

Author Affiliations

University of Manchester, Alliance Manchester Business School: Sarah Knowles PhD, Sarah Darley PhD

University of Manchester, Manchester Academic Health Sciences Centre (MAHSC), Manchester, UK:

- Division of Neuroscience and Experimental Psychology, School of Biological Sciences: Emma Patchwood PhD, Prof Audrey Bowen PhD, Claire Mitchell, PhD
- Division of Nursing, Midwifery and Social Work, School of Health Sciences: Prof Gunn Grande PhD
- Centre for Biostatistics, Division of Population Health Services Research and Primary Care, School of Health Sciences: Sarah Rhodes MSc

University of Cambridge, Centre for Family Research, Cambridge, UK: Gail Ewing PhD

Salford Royal Foundation Trust, Salford, UK: Kate Woodward-Nutt MSc

Manuscript word count (excluding title page, abstract, references, figures and tables): 4599
Abstract word count: 284
Tables: 3
Figures: 1

For peer review only

KEYWORDS
Stroke, Caregivers, Normalisation Process Theory, Process evaluation

ABSTRACT

Objectives
To examine the implementation of an intervention to support informal caregivers and to help understand findings from the Organising Support for Carers of Stroke Survivors (OSCARSS) cluster randomised controlled trial (cRCT).

Design
Longitudinal process evaluation using mixed methods. Normalisation Process Theory informed data collection and provided a sensitising framework for analysis.

Setting
Specialist stroke support services delivered primarily in the homes of informal carers of stroke survivors.

Participants
OSCARSS cRCT participants including carers, organisational staff, managers and senior leaders.

Intervention
The Carer Support Needs Assessment Tool for Stroke (CSNAT-Stroke) intervention is a staff-facilitated, carer-led approach to help identify, prioritise, and address support needs.

Results
We conducted qualitative interviews with: OSCARSS cRCT carer participants (11 intervention, 10 control), staff (12 intervention, 8 control), and managers and senior leaders (11); and obtained 140 responses to an online staff survey over three separate time points. Both individual (carer/staff) and organisational factors impacted implementation of the CSNAT-Stroke intervention and how it was received by carers. Staff valued the idea of a structured approach to supporting carers, but key elements of the intervention were not routinely delivered. Carers did not necessarily identify as “carers”, which made it difficult for staff to engage them in the intervention. Despite organisational enthusiasm for OSCARSS, staff in the intervention arm perceived support and training for implementation of CSNAT-Stroke as delivered primarily by the research team, with few opportunities for shared learning across the organisation.

Conclusions
We identified challenges across carer, staff, and organisation levels that help explain the OSCARSS cRCT outcome. Ensuring training is translated into practice and ongoing organisational support would be required for full implementation of this type of intervention, with emphasis on the carer-led aspects, including supporting carer self-identification.

Trial registration
OSCARSS cRCT ISRCTN58414120

ARTICLE SUMMARY

Strengths and limitations of this study

- Conducting a longitudinal process evaluation over the period of three years enabled us to ascertain how the intervention was implemented in practice and provide understanding of the neutral outcome of the trial.
- Mixed methods helped us capture the complexity of the intervention and allowed us to triangulate quantitative and qualitative data to increase validity and credibility of findings.
- We captured a range of perspectives from all participants in the trial, including staff implementing the intervention, carers receiving the intervention, senior managers and leaders within the organisation, and carers and staff within the control arm.
- Observation of implementation was not possible for practical reasons, which made intervention fidelity difficult to assess.
- We ran the process evaluation largely independent of the trial in order to establish effectiveness of the intervention under real world conditions. However, this meant that process information was not shared with the organisation until towards the end of the trial

For peer review only

FUNDING STATEMENT

This project was supported by the National Institute for Health Research Collaboration for Leadership in Applied Health Research and Care (NIHR CLAHRC) Greater Manchester and Stroke Association. NIHR had no role in study design, data collection, data analysis, data interpretation, or writing of the paper. Stroke Association partnered with NIHR in funding this study and was the specialist stroke service provider in OSCARSS. Some Stroke Association staff were research participants in this study and others contributed to discussions about findings and dissemination. The first author had full access to all the study data and had final responsibility for the decision to submit for publication. The views expressed in this article are those of the author(s) and not necessarily those of the NIHR, the Department of Health and Social Care, or the Stroke Association.

COMPETING INTERESTS

Dr. Grande reports grants from NIHR CLAHRC, during the conduct of the study; In addition, Dr. Grande has a patent Copyright issued.
Dr. Ewing reports grants from NIHR, during the conduct of the study; In addition, Dr. Ewing has a patent Copyright issued.
Mrs. Rhodes reports grants from NIHR, during the conduct of the study.
Prof. Bowen reports grants from NIHR, grants from Stroke Association, during the conduct of the study; grants from Stroke Association, grants from NIHR, outside the submitted work.
Dr. Patchwood reports grants from Stroke Association, outside the submitted work.
Dr Darley, Dr Knowles, Ms Woodward-Nutt and Dr Mitchell have nothing to disclose.

INTRODUCTION

Background

Informal caregivers, such as family members, play a significant role in providing daily support to stroke survivors who experience a range of life-limiting difficulties [1]. The caring role can be rewarding [2] but is also challenging with significant health implications [3], impacting on physical and emotional well-being, financial security, professional and social activities [4]. Current support for carers differs according to location but generally comes from hospital stroke units, local authority, specialist carer organisations, and stroke organisations [5].

Supporting informal carers is a statutory priority [6, 7, 8] but there is little clarity on how best to identify and assess the needs of people caring informally for stroke survivors [9, 10, 11]. One relevant approach is the Carer Support Needs Assessment Tool (CSNAT) intervention [12]: a comprehensive evidence-based assessment tool integrated within a multi-stage person-centred approach to individualised support. It was developed, implemented and tested in the context of palliative care with positive outcomes [13, 14, 15]. As one of the few available evidence based interventions for carers that are carer-led and that enable comprehensive assessment and support, the CSNAT was considered worth exploring for supporting informal carers of people with long-term health conditions. For the present study, the CSNAT intervention was adapted for use in stroke care, through collaboration with a specialist stroke organisation and a group of lay carers. The involvement of carers in particular was integral in making the wording stroke specific and informing implementation of the intervention in practice [16]. The adaptation resulted in the CSNAT-Stroke intervention, including a staff training and implementation package tailored to the stroke organisation. In brief, the intervention is a person-centred, structured process of needs assessment and support that is practitioner-facilitated, but carer-led, comprised of a four-stage process, including a needs assessment tool and action plan (see Table 1).

Table 1 CSNAT-Stroke intervention as intended in OSCARSS

Key details of the intervention using descriptors from the TIDieR checklist [17], adapted from Patchwood et al. [paper submitted]

Item	CSNAT-Stroke intervention, as intended for implementation within the stroke organisation
Why	To provide a person-centred process of assessment and support for carers that is practitioner-facilitated but carer-led and tailored to the carer's individual needs, which are likely to change over time. The intervention assumes that carers may have difficulty considering and expressing their needs.

For peer review only - http://bmjopen.bmj.com/site/about/guidelines.xhtml

What (materials and procedures)	Materials / procedures include: (A) four-stage process (components outlined below), (B) a needs assessment tool, (C) an action plan.<br>**1. Identifying and introducing:**<br>Staff identify the carer early and make clear that support is available ('scripts' for sensitive use of the term "carer"). Staff introduce needs assessment tool to carers during separate time with the carer, providing an opportunity to discuss support needs.<br>**2. Needs assessment**<br>Carers are given time to consider their support needs, self-completing the tool, identifying the areas in which they need more support and prioritising those most important to them. Staff normalise the practice of having separate time with the carer as well as stroke survivor if present, to support each as individuals.<br>**3. Assessment conversation and tailoring**<br>An assessment conversation between carer and stroke practitioners identifies the carer's individual support needs and what they feel would be most supportive within the domains of the needs assessment tool that they have prioritised. Support may be directly delivered by staff at this time (e.g. active listening, information, signposting/referrals) but helping carers identify sources of support they may wish to access themselves (self-help) or via family members and friends is also encouraged. Staff create service directories to support signposting/referral.<br>**4. Shared plan for action and review**<br>Carers record a plan of supportive input describing the actions taken or to be taken by the practitioner or carer to address identified needs, which will be subsequently reviewed as appropriate.<br>Each stage should be staff-facilitated but carer-led. At all stages the carer should be given the opportunity to say what is most important to them and what they feel would help support them.
Who provided	Frontline staff: Essential criteria for recruitment to this role includes GCSE education, experience providing care to vulnerable people, and good communication skills. Training in stroke and stroke-specific care is provided by the organisation. Additional intervention specific training provided to frontline staff in clusters allocated to intervention.<br>Training, co-delivered by the service provider and the research team, is with groups of staff over a half-day session involving instructional videos and scripts, role-play, and workbook completion.
How	After training the intervention should be implemented by staff during their routine support visits.
Where	Typically in carers' homes.
When and How much	The intervention should be used every time a staff member has contact with a carer and requires a minimum of 1 face-to-face support contact with the carer, with reviews likely.
Tailoring and modifications	Staff training is modified for those joining after the primary roll-out of the intervention and adapted to one-to-one delivery by the research team using video conferencing.

Organising Support for Carers of Stroke Survivors (OSCARSS) was a cluster randomised controlled trial (cRCT) which aimed to investigate the clinical- and cost-effectiveness of the CSNAT-Stroke intervention for supporting carers relative to a control of usual practice within the stroke organisation [18]. OSCARSS found no meaningful differences in self-reported clinical outcomes for carers, such as strain and distress, between intervention and control groups [paper submitted]. This paper presents findings from the embedded process evaluation of OSCARSS, which aimed to enrich understanding of the trial result, understand the factors influencing implementation and acceptability of a carer-led intervention, and consider implications for future research and practice.

For peer review only

METHODOLOGICAL APPROACH

Process evaluations provide insight into the 'black box' of implementation of a complex intervention [19] and help explain discrepancies between expected and observed outcomes within a context [20]. The process evaluation for OSCARSS used longitudinal mixed methods to examine ongoing experiences of and perspectives on both usual practice and the CSNAT-Stroke intervention from staff within the stroke organisation, as well as carers who receive their support and consented to join OSCARSS.

Normalisation Process Theory (NPT) was used as a framework to guide data collection and analysis, and sensitised us to certain points in the data for both agreement and resistance [21]. NPT is a commonly used framework in process evaluations of complex interventions in healthcare because it increases understanding of the dynamic processes involved during their implementation and helps to explain mechanisms that affect their outcomes [22]. NPT considers both agency (individual factors) and context (organisational factors) and proposes four constructs to explain how interventions are embedded into practice. Constructs include: Coherence (sense-making), Cognitive Participation (engagement and enrollment), Collective Action (how the work happens in existing systems), and Reflexive Monitoring (evaluation) [23].

SETTING AND SAMPLE

The OSCARSS cRCT ran from January 2017 to December 2018. It recruited 414 carers over an 18 month recruitment period from 35 randomised clusters made up of the stroke organisation's services across England and Northern Ireland (18 intervention and 17 control). Approximately 100 frontline staff who were employed at participating clusters during the study period were trained to participate in OSCARSS. Frontline staff are non-clinical support staff that provide personalised information, advice and support to stroke survivors and their carers.

The process evaluation purposefully sampled a wide range of participating frontline cluster staff and consented carers from both trial arms. Staff, managers and senior leaders were approached to participate by email; carers were approached by telephone if they consented to be contacted for this purpose during initial trial recruitment.

METHODS

We used mixed methods including training observations, qualitative interviews, and online questionnaires at different time points to capture the perspectives of different stakeholders during the course of the study (Figure 1). Triangulating methods and incorporating multiple perspectives and experiences aimed to increase the credibility and trustworthiness of our findings [24]. Ethics approval was obtained from the North West Lancaster Research Ethics Committee (ref: 16/NW/0657) and University of Manchester (ref: AMBS-2016-22). All participants gave informed consent.

Qualitative semi-structured interviews were conducted to provide rich descriptions of experiences and perspectives of both carers and staff. We conducted telephone interviews with carers as soon as possible after joining the main trial. Carer interviews were completed by members of the trial team (EP, KWN) and an experienced qualitative researcher (CM) between May 2017 and June 2018. Each carer was interviewed at the point of study consent and, if they agreed, again after primary outcomes had been collected at three months. Topic guides (supplementary material 1) were informed by the research aim and feedback from the RUG, and explored carers' experiences of support and their perceptions of the impact of support received.

Staff interviews were conducted with cluster frontline staff, managers and senior leaders between August and November 2018. All staff interviews were conducted by SD who had not been involved with staff training or support. Staff interviews were conducted face-to-face or over the telephone, depending on staff location and preference. Interview topic guides (supplementary material 2) were informed by NPT and aimed to capture context and carer support practices, and strategic organisational priorities (relevant for managers and senior leadership). For intervention-allocated frontline staff, additional questions explored changes to practice, mechanisms that supported changes, and perceived outcome of changes to practice. Researchers made field notes and reflections after all interviews, which lasted between 30-60 minutes. Interviews were audio recorded with anonymised transcripts, field notes, and reflections uploaded to Nvivo software to facilitate thematic analysis.

For peer review only

Online questionnaires were distributed via email to all frontline staff involved in OSCARSS at three separate time points: T1 pre-randomisation of clusters in August 2016, T2 and T3 post-randomisation in August 2017 and July 2018 respectively. Questionnaires asked about existing practice, attitudes towards supporting carers, the wider context within which staff worked, and demographic information. At T2 and T3, the questionnaire for intervention arm staff additionally included items based on the NOrmalization MeAsure Development (NoMAD) [25] (supplementary material 3). NoMAD contains Likert items designed to explore intervention implementation, based on NPT.

Staff training in the intervention arm was typically delivered by the stroke organisation over half day group sessions and observed by KWN and EP. Field notes were taken to capture: perceived engagement of attendees with the training; whether all components of the training had been delivered as intended; types of questions asked by attendees and responses given. Staff also completed training feedback forms to explore their understanding of key components of the intervention.

PATIENT, CARER, AND PUBLIC INVOLVEMENT (PCPI)

A study specific Research User Group (RUG) of ten individuals with experience of caring for a stroke survivor was set up in December 2015, at the planning stages of OSCARSS. Through regular meetings (2015-2019) and representation on the Trial Management Group the priorities, experiences, and preferences of the RUG informed development of the research questions and the design, analysis, and dissemination of both the trial and process evaluation. The RUG advised on participant recruitment and were central in limiting the burden of participation for carers. The RUG also supported adaptation of the CSNAT intervention and staff training package, including role-playing videos of the intervention in practice. A video summarising their role in OSCARSS is available on the study website [https://www.arc-gm.nihr.ac.uk/projects/oscarss] and a paper reflecting on their input [16]. In the early stages of data analysis three meetings were held to focus on the process evaluation where SD, SK and EP shared and discussed themes from the data with the RUG. In these meetings, NPT and the value of this framework for understanding the findings were collaboratively discussed. The RUG helped us consider rival explanations, new ideas, and implications on practice.

DATA ANALYSIS

Qualitative data

Interview data were coded inductively by several members of the research team (CM, EP, KWN, SD) to develop a coding framework. This framework was further developed by process evaluation leads (SD and SK) who coded the data deductively guided by the NPT framework. Analysis was an iterative process and the combination of inductive and deductive approaches allowed codes to fit into a structure, relate to each other in meaningful, study-important ways [26] and enabled identification of prominent themes. Other relevant data (e.g. training materials, observation field notes, and staff training feedback questionnaires) were reviewed throughout analysis (by SD and SK) to provide further context.

Quantitative data

Questionnaire data were analysed descriptively to provide summary information on usual practice of supporting carers within the stroke organisation and included NoMAD items relating to use and acceptability of the intervention.

Data synthesis

All data were synthesised and thematically analysed using the four main constructs of NPT [23] to identify patterns across the data [27]. As in previous studies this process allowed us to combine qualitative and quantitative findings [28] into one explanatory framework [26]. Researchers experienced in NPT (SD and SK) led this method of data analysis following a continuous, iterative process of reviewing and refining themes through discussions with the research team and RUG. We analysed at two levels: the individual and the organisation.

Draft findings were presented to multiple audiences, including internal PCPI collaborators (the RUG), Trial Management teams, and at external conferences and seminars. This enabled input from a multiplicity of viewpoints and supported reflective discussions to reach consensus on core, prominent themes.

RESULTS

Participants

For peer review only

Twenty-one carers of stroke survivors participated in first interviews (11 intervention, 10 control) and 11 agreed to follow up interviews (4 intervention, 7 control) (Table 2). Carers typically declined interviews when they felt they had nothing new to add.

Table 2 Carer characteristics

Key

SS needs score = independence level as reported by Carer (Min score = 1, Max score= 3)
C= control arm, I= intervention arm
M = Male, F = Female

Allocation	Gender	Age	Relation to Stroke Survivor	Stroke date	Ethnicity	SS needs score	Days from consent to interview	Status (1st/2nd interviews)
I	M	50-59	Partner	Feb 2017	White	2	33	completed 1&2
I	F	50-59	Daughter	May 2017	White	2.7	28	completed 1&2
I	M	60-69	Partner	May 2017	Asian	3	10	completed 1&2
I	F	60-69	Partner	Jan 2017	White	2.7	18	completed 1&2
I	F	30-39	Daughter	Jan 2017	Asian	1.7	4	opted out 2
I	F	50-59	Daughter	Aug 2017	White	3	21	opted out 2
I	F	50-59	Partner	June 2017	White	1	3	opted out 2
I	F	70-79	Partner	March 2017	White	1.7	19	opted out 2
I	M	70-79	Friend	March 2018	White	2	28	opted out 2
I	M	70-79	Partner	June 2017	White	1	29	opted out 2
I	F	70-79	Partner	Nov 2017	White other	1	65	opted out 2
C	F	40-49	Partner	Nov 2017	White	1	16	completed 1&2
C	F	60-69	Partner	Jan 2017	White	3	29	completed 1&2
C	M	60-69	Partner	Feb 2017	White	1.8	20	completed 1&2
C	M	60-69	Partner	Nov 2017	White	2.7	23	completed 1&2
C	F	60-69	Partner	Jan 2018	White	1.8	17	completed 1&2
C	F	70-79	Partner	Feb 2018	White	2.2	29	completed 1&2
C	F	70-79	Partner	March 2017	White	3	14	completed 1&2
C	F	60-69	Parent	July 2016	White	1.8	20	opted out 2
C	F	50-59	Daughter	Sep 2017	White	2.7	77	opted out 2
C	F	50-59	Daughter	Dec 2017	White	1.6	46	opted out 2

Thirty-one staff members were interviewed (frontline staff: 12 intervention, 8 control; managers and senior leaders across clusters: 11). Thirteen additional frontline staff declined interviews. Staff participants were based across England and Northern Ireland, included both full and part-time workers and length of service ranged from less than a year to 23 years.

We observed 25 hours of staff training on the CSNAT-Stroke intervention over six group sessions, including 47 intervention-allocated staff. Response rates to the online staff questionnaire were: T1 72% (62 pre-intervention), T2 48% (20 intervention, 21 control), T3 35% (20 intervention, 17 control). Due to staff turnover, the same staff did not necessarily answer each questionnaire. Questionnaire respondents were predominantly female, aged 43 and over, worked part-time, and length of service ranged from less than one year to over ten.

For peer review only

Themes

In this section, we draw upon themes identified through the NPT framework to report factors that impacted on implementation of the intervention (Table 3). For this purpose we mainly present data from the staff intervention arm (interviews and questionnaires T2 and T3), as well as from carers, managers and senior leaders.

Table 3 Themes identified through the NPT framework

NPT construct and definition	Relevant questions considered to guide analysis	Theme from data
**Coherence:** Understanding and valuing of the intervention	Was the intervention understood as different from usual practice?<br>What were the beliefs and behaviours that defined the new approach?<br>Was there a shared understanding of the intervention?<br>Was there a benefit identified in following the intervention?	**Limited understanding of different components of the intervention:** Although all staff valued the ethos of a carer-led approach to supporting carers, despite the training staff demonstrated a limited understanding of the whole intervention.
**Cognitive Participation:** Engagement in and activation of the intervention by individuals and groups	How was the intervention initiated?<br>Did staff and carers feel like legitimate participants in the intervention?<br>How were staff and carers enrolled and engaged in the intervention?<br>What promoted or inhibited participation in the intervention?	**Limited participation in the intervention:** Staff engaged with the intervention but struggled to persuade carers to participate and carers did not necessarily see themselves as legitimate participants.
**Collective Action:** How participants work to carry out the intervention	How was the intervention carried out?<br>What were the beliefs and behaviours of staff and carers that define and organise the work?<br>How did staff fit the new approach into their existing practice and context?<br>Who was best placed to make use of the intervention?	**The intervention was not fully implemented:** Key components of the approach (such as a separate conversation between carer and staff member, and completion of the action plan) were not always implemented.
**Reflexive Monitoring:** How the intervention is appraised and evaluated	What factors promoted or inhibited appraisal of the intervention?<br>How was the intervention monitored and evaluated?<br>How did staff and carers appraise the practice?<br>Was the intervention modified or adapted?	**Limited opportunities to share learning and ongoing support for the intervention:** All staff positively appraised the intervention as they understood it, but there were limited opportunities to share learning and receive ongoing support from across the stroke organisation.

We present our findings by focusing at the individual staff and carer level (covering themes of coherence and cognitive participation) and then at the organisational level (covering themes of collective action and reflexive monitoring). Using the NPT framework to take both levels into account helps identify the individual behavioural changes and agency, as well as the organisational structural and cultural changes required to implement a complex intervention [22].

Individual staff and carer level factors (Coherence and Cognitive Participation)

For peer review only

Carers in both arms of the study reported doing a wide range of caring tasks including personal care, practical tasks, and financial administration. They described the impact of caring on their emotional and physical wellbeing, restricted social life, having to give up work, and financial implications:

> "I'm 77 and it is hard work… it doesn't switch off." [Carer 1, intervention, interview 1]

However, during support visits carers did not always feel able to consider their own needs as their priority was the person they were caring for. This inhibited their **Cognitive Participation** in the intervention. Frontline staff saw this as a persistent issue, despite training for the intervention that included how to introduce CSNAT-Stroke during initial contact in a way that was sensitive to carer self-identification:

> "Where there's sometimes debate is when you're labelling someone as a carer and often someone's wife, for example, would say - I'm not his carer, I'm his wife." [Frontline staff member 1, intervention, interview]

Carers in the control arm appraised the support they received from the stroke organisation just as positively as carers did in the intervention arm, particularly when they received little support from other organisations. Generally all carers interviewed said that they were grateful to have somebody interested in their needs and able to provide practical help.

More than half of intervention arm staff respondents to T2 and T3 questionnaires said they could see how the intervention differed from their usual way of working and could be easily integrated into current practice. However, in the interviews where this issue was explored in detail there were mixed perceptions of the difference, particularly for staff who had been in the role for a number of years:

> "I'd say it was quite similar, it's just the way it's outlaid isn't it really? What I do is not on paper as such… I've got experience behind me and I've worked with carers for a long time." [Frontline staff member 2, intervention, interview]

The desire for a structured, formalised approach to offering carer support came through strongly from all staff, including senior leaders. Over half the intervention respondents to T2 and T3 agreed that staff in their service had a shared understanding of the intervention. However, the intervention was primarily perceived as solely the paper-based assessment needs tool:

> "It's been a real pleasure to use, I consider it useful …in the beginning it was like okay great, more paper, but actually it's one of my most useful pieces of paper and I've got a lot of paper." [Frontline staff member 3, intervention, interview]

The interviews helped identify the lack of **Coherence** of the intervention through descriptions by staff of not fully implementing it and the similar accounts of support received by carers in both trial arms. Staff also appeared to see certain parts of the intervention, such as the action plan, as duplicating existing practice rather than a route to empowering carers to take ownership of actions for themselves:

> "I have to confess, I didn't [use the action plan]… I would follow it up in a letter … I don't see the point in doing [the action plan] …I'd be duplicating it." [Frontline staff member 4, intervention, interview]

Frontline staff positively appraised the OSCARSS training and, in feedback forms, highly endorsed that they understood each stage of the intervention and felt confident to deliver it. Despite this, interviews suggested that the training and ongoing support had not been sufficient to equip staff with a full understanding of the carer-led process of the intervention. In particular, the initiation of a separate conversation with a carer to discuss their needs is an important part of the intervention that was championed by the RUG. Observation notes showed that this point was often debated in the group training sessions, with some staff anticipating difficulty in talking with carers separately to stroke survivors. Staff described that historically these separate conversations tended to be intermittent and initiated by carers (often on the doorstep at the end of a visit), whereas OSCARSS training encouraged staff to routinely initiate a separate conversation and normalise this in advance of a visit. Whilst staff saw the value of this in principle, they did not always initiate it in practice and instead many reported asking carers for the first time about meeting separately during the visit and in front of the stroke survivor:

> "What I find is I'll introduce it but people don't want to go off and talk on their own. They want to stay with the stroke survivor. Some people do, but some people don't. They said, oh no, that's fine, we can talk quite openly with each other, so you can't make people go and talk on their own." [Frontline staff member 5, intervention. interview]

The lack of full implementation was also evident in the needs assessment process. Staff would often assume what carers' needs might be and anticipate not being able to meet them, which led to a reluctance to comprehensively discuss needs:

For peer review only

> "To sort of say, okay, I recognise you're struggling with that and I don't have a solution… you're perceiving them to think, what was the point of asking me if I needed help - you've just highlighted the fact I need help and there's no services to help me." [Frontline staff member 1, intervention, interview]

Frontline staff had a well-meaning, strong desire to practically meet carer needs and 'fix' problems. However, core tenets of the carer-led approach of the intervention were that needs should be heard to normalise needs and permit collaborative problem-solving, tailoring support to the carer's priorities.

Despite the lack of full implementation, components of the intervention that were understood were positively appraised by individual staff. For example, many expressed a desire to continue using the needs assessment tool in their meetings with carers beyond the trial. Managers were also keen to continue a systematic, structured approach to supporting carers.

The organisational level and contextual factors (Collective Action and Reflexive Monitoring)

The focus on carers and the carer-led approach of the intervention aligned with organisational values and was strongly supported by senior leaders and managers. However, managers did not have a comprehensive understanding of the intervention and whilst frontline staff perception of management support for the intervention increased over time, they saw training for and communications about the intervention as coming from the research team rather than the organisation:

> "I don't think really the [organisation] got involved much 'cause it was separate, wasn't it? Some [services] were included, some weren't, so I think they just left [the research team] to it and let you support us." [Frontline staff member 2, intervention, interview]

Frontline staff were viewed as most appropriate to deliver the intervention through **Collective Action**, although over half of T2 and T3 respondents in the intervention arm were neutral on whether work was assigned to those with skills appropriate to the intervention. At T2 and T3 frontline staff expressed openness to working with colleagues in new ways to use the intervention and in interviews frontline staff expressed a desire to learn from or share experiences with other teams.

However, there appeared to be few opportunities for shared learning on the intervention. Staff usually worked from home and did not have regular contact with each other, so opportunities were unlikely to happen without managerial planning. **Reflexive Monitoring** was limited with no systematic monitoring or evaluation of the intervention within the organisation, although almost all of T2 and T3 intervention respondents agreed that feedback could be used to improve the intervention.

During OSCARSS, services across the organisation experienced significant reorganisation internal staffing changes and commissioning changes. In interviews some frontline staff described difficulty participating in the intervention within the context of internal and external changes and pressures:

> "OSCARSS hasn't been a priority at all, we've been going through a lot of changes, we've lost some of our funding, we've had commissioned services being cut. There has been much more priority than doing the trial." [Frontline staff member 6, intervention, interview]

Just over half T2 and T3 intervention respondents agreed or strongly agreed that sufficient resources were available to support the intervention. However, in staff interviews a lack of time for and during visits to stroke survivors and carers was highlighted as inhibiting participation in the intervention:

> "I think time would be the main thing, because… a lot of [frontline staff] just wouldn't necessarily factor the time in to a visit because they're already quite long, and there is already quite a lot of things to do within that visit." [Senior Leader, interview]

DISCUSSION

The OSCARSS process evaluation helps us understand the context in which the intervention was delivered within the trial and provides some explanation of why no meaningful added benefit was evident when comparing intervention with usual practice. We identified significant challenges to successful implementation of the intervention at both individual and organisational levels. Frontline staff were mainly positive about the intervention, insofar as they wished to enhance carer support, but they did not have a full understanding of the

For peer review only

carer-led aspects of the intervention. As in previous studies [29] this lack of coherence was a key factor in the intervention not being fully implemented. The training that frontline staff received through OSCARSS (half a day face-to-face or 1-3 hours over Skype) and ongoing informal support from the research team was not sufficient to provide this understanding. Frontline staff cared deeply about supporting carers, but their tendency to anticipate and 'fix' needs limited opportunities for the approach to be carer-led. Key stages of the intervention were also not routinely implemented, such as a separate conversation and completion of an action plan. Consistent with this finding, interviews with carers demonstrated little difference in support provided across the trial's intervention and control arms. Despite positive engagement and organisational enthusiasm for OSCARSS, the intervention was not delivered as intended. The findings demonstrate challenges to delivery of carer-led assessment and support, including overcoming the reluctance of carers to express their own needs, frontline staff anticipating carer needs, and a need for ongoing organisational support and learning.

Although there is strong evidence for the need to support carers of stroke survivors [30, 31] and a statutory requirement to do so [8] those considering implementing carer-led interventions should be aware of the challenges and solutions highlighted by OSCARSS. Carers in our study described the range of tasks they were responsible for in providing care and the impact this has on their lives. However, in line with other studies the carers did not always feel ready to accept support for themselves and did not necessarily see themselves as "carers" [32, 33] and the intervention was not implemented in a way to address this issue. Using NPT emphasised that motivation and 'buy in' for an intervention relies on engagement of both practitioner and service recipient to 'enroll' themselves as eligible and appropriate for an intervention to work. This may be particularly challenging for carers. Our findings contribute to this often overlooked yet vital aspect in implementation theory [34] and our multi-stakeholder analysis enabled us to explore both individual and wider contextual factors that impact on implementation in practice.

Our process evaluation has shown the difficulty implementing a complex intervention that follows a carer-led ethos, despite strong organisational enthusiasm and staff willingness to support carers. Our findings show that staff would have welcomed additional ongoing support and shared learning opportunities to gain a full understanding of the carer-led approach. Support mechanisms used in previous CSNAT intervention implementations, such as developing staff champions [35, 36] may have ensured that managerial commitment was communicated to frontline staff. However this was difficult within the design of the cluster RCT as in some services a cluster may have only included one or two staff members. The trial team tried to offer similar support and championing to intervention arm frontline staff, but our findings indicate that such support would have been more effective coming from the organisation itself, Many carers across both trial arms said that frontline staff in the organisation were one of their main sources of support and this was highly valued. In OSCARSS, the intervention was developed to be used within the pragmatic organisational context of individual staff supporting both stroke survivor and carer, requiring no additional resources. However, our findings highlight the barriers for coordinators to fully support both individuals within their available resources and limited time. Our findings also highlight the particular complexity of meeting needs of carers who struggle to recognise their own needs, and the need to consider timing of support and how to sensitively and appropriately facilitate conversations with carers and stroke survivors.

The process evaluation was led by experienced researchers separate to the trial team and used multiple methods over time to generate rich and in-depth data. It used NPT to bring together these data that provided a range of perspectives at different time points and sampled from both trial arms across clusters. Through examining implementation at the individual and organisational level, the process evaluation offers insight into why the intervention was not fully implemented and contributes to knowledge on implementing carer-led approaches in health care. A limitation to the study was that observation of the intervention in real-time was not conducted. Reasons for this included the difficulty of minimising Hawthorne effects on staff implementing the intervention, ethical considerations of carers discussing sensitive information at a potentially distressing time, and practicalities of observation within this large-scale study of 35 clusters. This limitation impacted on the extent to which we could assess fidelity as we were not able to triangulate interview and question data with observations of practice. However, we recruited carers and staff from different geographical areas within the trial and triangulated multiple data to get an understanding of implementation from the perspective of several stakeholder groups. The process evaluation was also ran largely independent of the trial in order to establish effectiveness of the intervention under real world conditions [20]. However, this resulted in sharing process information with the organisation only towards the end of the trial, meaning that additional strategies to help support full implementation of the intervention were not explored. In hindsight, taking into account our findings, it may have been useful to have planned the process evaluation in stages with an early review, and also run an initial feasibility study before the full trial.

The process evaluation showed that the intervention was not fully implemented as intended, therefore we cannot say whether it may provide a benefit to carers of stroke survivors. Using NPT we have highlighted areas that need to be addressed in future research of this intervention or similar person-led approaches to supporting carers

For peer review only

of stroke survivors. Considerations include firstly, how to train and support staff, including strategies to help with engaging carers reluctant to prioritise their own need. Secondly, ensuring clear communication pathways to ensure adoption of the intervention, involving management goals top down to frontline staff and also bottom up enabling practitioners to raise implementation difficulties and have them resolved. Thirdly, having a network of staff champions as role models and for problem solving. Fourthly, clarity at the outset about resources needed for implementation and sustainability.

CONCLUSIONS

This paper shows the value of process evaluation in helping to interpret neutral trial results, such as OSCARSS. It demonstrates how both staff and service user perceptions and organisational demands can impede the delivery of key intervention components, even with significant buy-in to the need for the intervention itself. Our findings identified certain challenges, as well as opportunities, that contribute to knowledge on implementing carer-led support interventions. We recommend that future intervention work should concentrate on addressing organisational factors which can both help and hinder implementation and that all levels within the organisation are engaged in implementation, including undertaking training to have a full understanding of all components of the intervention.

AUTHOR CONTRIBUTIONS

All authors (SD, SK, KWN, CM, GG, GE, SR, AB, EP) contributed to the design of this study. SD, KWN, CM and EP contributed to recruitment, collected the data and contributed to the analysis. All authors (SD, SK, KWN, CM, GG, GE, SR, AB, EP) contributed to interpretation of findings and writing of the manuscript. Jessica Stolberg contributed to collection of questionnaire data and Verity Longley contributed to analysis. The trial steering committee contributed to the study design and interpretation of findings. The study specific carer advisory research group contributed to the study design and interpretation of findings: Kelly Burke, Christine Halford, Natalie Halford, Geoff Heathcote, Kath Purcell and Ben Wright.

ACKNOWLEDGEMENTS

The authors would like to thank all of the carers and staff members who participated and contributed to this study. We would like to thank members of the supportive NIHR CLARHC GM team who helped make the study possible including: Caroline O'Donnell (data analyst); Alison Littlewood and Katy Rothwell (programme managers); Sam Wilkinson, Amy Woodhouse and Rose Crees (administrative assistants); Aneela Macavoy and Zoe Ashton (research facilitators). We also wish to thank and acknowledge Stroke Association staff, including Chris Larkin, Elaine Roberts, Cass Saunders and Kathryn Staley.

DATA SHARING STATEMENT

All data relevant to the study are included in the article or uploaded as supplementary information. No additional data available.

REFERENCES

1 Crichton SL, Bray BD, McKevitt C, *et al.* Patient outcomes up to 15 years after stroke: survival, disability, quality of life, cognition and mental health. *J Neurol Neurosurg Psychiatry Res*, 2016;87:1091–1098.

2 Mackenzie A, Greenwood N. Positive experiences of caregiving in stroke: a systematic review. *Disabil Rehabil*. 2012;34(17):1413-1422.

3 Noonan MC, Wingham J, Taylor RS. 'Who Cares?' The experiences of caregivers of adults living with heart failure, chronic obstructive pulmonary disease and coronary artery disease: a mixed methods systematic review *BMJ Open,* 2018;8:e020927.

4 Quinn K, Murray C, Malone C. Spousal experiences of coping with and adapting to caregiving for a partner who has a stroke: a meta-synthesis of qualitative research. *J Disabil Rehabil,* 2014;36:185–198.

5 The Stroke Association. Stroke: a carer's guide. 2018. Available: https://www.stroke.org.uk/sites/default/files/media-root/f04_stroke_a_carers_guide_v2.2.pdf [accessed 18/06/2020]

For peer review only

6 Carers UK. NHS England's Long Term Plan and what it means for carers, 2019. Available: https://www.carersuk.org/for-professionals/policy/policy-library/the-nhs-long-term-plan-and-what-it-means-for-carers [accessed 12 August 2019]

7 Department of Health & Social Care. Carers Action Plan 2018 to 2020: Supporting carers today. Available www.nationalarchives.gov.uk/doc/open-government-licence/ [accessed 6 September 2019]

8 Department of Health, Care Act. 2014. Available: http://www.legislation.gov.uk/ukpga/2014/23/contents/enacted/data.htm [accessed 21 November 2019]

9 Ellis G, Mant J, Langhorne P. *et al.* Stroke liaison workers for stroke patients and carers: an individual patient data meta-analysis. *Cochrane Database Syst Rev.* 2010; 2010(5):CD005066.

10 Legg LA, Quinn TJ, Mahmood F. *et al.* Non-pharmacological interventions for caregivers of stroke survivors. *Cochrane Database Syst Rev*. 2011;(10):CD008179.

11 Forster A, Brown L, Smith J, *et al.* Information provision for stroke patients and their caregivers. *Cochrane Database Syst Rev*. 2012;11(11):CD001919.

12 Ewing G, Grande GA. Development of a Carer Support Needs Assessment Tool (CSNAT) for end-of-life care practice at home: *Palliat Med,* 2012;27:244-256.

13 Ewing G, Austin L, Grande G. The role of the Carer Support Needs Assessment Tool in palliative home care: A qualitative study of practitioners' perspectives of its impact and mechanisms of action. *Palliat Med*, 2016;30:392–400.

14 Ewing G, Brundle C, Payne S. The Carer Support Needs Assessment Tool (CSNAT) for Use in Palliative and End-of-life Care at Home: A Validation Study. *J Pain Symptom Manage*, 2013;46:395-405.

15 Aoun SM, Grande G, Howting D, *et al.* The impact of the carer support needs assessment tool (CSNAT) in community palliative care using a stepped wedge cluster trial, *PLoS One*, 2015;10:4.

16 Mitchell C, Burke K, Halford N, *et al.* Value and learning from carer involvement in a cluster randomised controlled trial and process evaluation - Organising Support for Carers of Stroke Survivors (OSCARSS). *Res Involv Engagem* 2020;6:21.

17. Hoffman TC, Glasziou PP, Boutron I, *et al.* Better reporting of interventions: template for intervention description and replication (TIDieR) checklist and guide, *BMJ*, 2014;348:g1687

18 Patchwood E, Rothwell K, Rhodes S, *et al.* Organising Support for Carers of Stroke Survivors (OSCARSS): study protocol for a cluster randomised controlled trial, including health economic analysis. *Trials*, 2019;20:19.

19 Grant A, Treweek S, Dreischulte T, *et al.* Process evaluations for cluster-randomised trials of complex interventions: a proposed framework for design and reporting. *Trials*, 2013;14:15.

20 Moore GF, Audrey S, Barker M, *et al.* Process evaluation of complex interventions: Medical Research Council guidance. *BMJ*, 2015;350:h1258.

21 Mills MR, Bettis PJ. Organizational Identity and Identification During a Departmental Reorganization. In: Anfara VA, Mertz NT, eds. Theoretical Frameworks in Qualitative Research. London: Sage 2006:73-84.

22 May CR, Cummings A, Girling M, *et al.* Using Normalization Process Theory in feasibility studies and process evaluations of complex healthcare interventions: a systematic review. *Implement Sci*, 2018;13:80.

23 May C, Finch T. Implementing, Embedding, and Integrating Practices: An Outline of Normalization Process Theory. *Sociology*, 2009;43:535–554.

24 Patton M. *Qualitative evaluation and research methods*. Beverly Hills, CA: Sage 1990.

25 May CR, Finch T, Ballini L, *et al.* Evaluating complex interventions and health technologies using normalization process theory: development of a simplified approach and web-enabled toolkit. *BMC Health Serv Res*, 2011;11:245

26 Moran-Ellis J, Alexander VD, Cronin A, *et al.* Triangulation and integration: Processes, claims and implications. *Qual Res*, 2006;6:45–59.

27 Braun V, Clarke V. Using thematic analysis in psychology, *Qual Res Psychol,* 2006;3:77-101.

For peer review only

28 Lewis NV, Dowrick A, Sohal A, *et al.* Implementation of the Identification and Referral to Improve Safety programme for patients with experience of domestic violence and abuse: A theory-based mixed-method process evaluation. *Health Soc Care Community*, 2019;27:e298-e312.

29 Lloyd A, Joseph-Williams N, Edwards A, *et al.* Patchy 'coherence': using normalization process theory to evaluate a multi-faceted shared decision making implementation program (MAGIC). *Implementation Sci*, 2013;8: 102

30 Haley WE, Roth DL, Hovater M, *et al.* Long-term impact of stroke on family caregiver well-being: a population-based case-control study. *Neurology*, 2015;84:1323–1329.

31 Kruithof WJ, Post MWM, Van Mierlo ML, *et al.* Caregiver burden and emotional problems in partners of stroke patients at two months and one year post-stroke: Determinants and prediction. *Patient Educ Couns*, 2016;99: 1632–1640.

32 Knowles S, Combs R, Kirk S, *et al.* Hidden caring, hidden carers? Exploring the experience of carers for people with long-term conditions*. Health Soc Care Community*, 2016;24:203–213.

33 Cameron JI, Gignac MAM. "Timing It Right": A conceptual framework for addressing the support needs of family caregivers to stroke survivors from the hospital to the home. *Patient Educ Couns*, 2008;70:305–314.

34 Kennedy A, Rogers A, Chew-Graham C, *et al.* Implementation of a self-management support approach (WISE) across a health system: a process evaluation explaining what did and did not work for organisations, clinicians and patients. *Implement Sci*, 2014;9:129.

35 Diffin J, Ewing G, Harvey G, *et al.* Facilitating successful implementation of a person-centred intervention to support family carers within palliative care: a qualitative study of the Carer Support Needs Assessment Tool (CSNAT) intervention. *BMC Palliat Care*, 2018;17:129.

36 Austin L, Ewing G, Grande, G. Factors influencing practitioner adoption of carer-led assessment in palliative homecare: A qualitative study of the use of the Carer Support Needs Assessment Tool (CSNAT), *PLoS One*, 2017;12:6.

FIGURES

Figure 1: Process evaluation methods

(Submitted as a separate file.)

This figure shows how the mixed methods used within the process evaluation were conducted alongside the cRCT

For peer review only

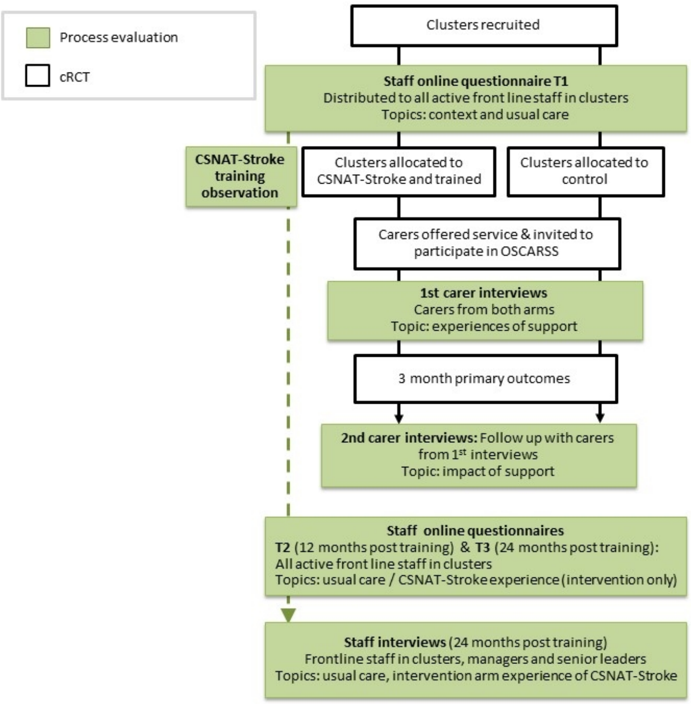

Figure 1 Process evaluation methods overview within the OSCARSS trial

254x190mm (96 x 96 DPI)

SUPPLEMENTARY MATERIAL 1

Topic guides for carer interviews

First interviews: Questions about experiences of support

I'd like to speak to you about contact you've had from [stroke organisation].

- Can you talk me through what happened during the visit?
  - Did you talk with X on your own or with others? (how was that / how did that go?)
  - Was any paperwork used? Possible additional probs : Can you tell me more about the paperwork you used? How was that / how did that go?
  - Did you keep a copy of any paperwork? If so, do you ever refer to it / find it helpful?
  - Did you talk about any support that you might need , as someone who is providing care/ support to <INSERT RELATIONSHIP eg mum >> How was that / How did that go?
  - Did you feel that you or the staff member 'took the lead' when talking? (Could re-visit 'paperwork' prompts here too)
- Did you come up with ideas of how to address the needs/issues you talked about? Can you talk me through them ?
  - Depending on if/how addressed earlier could repeat: Did you feel that you or the coordinator 'took the lead' when coming up with ideas?
  - Were you happy with the solutions/ ideas for support?
  - Were there any things that are not being addressed/ that it wasn't possible to address? What were these? How do you feel about this?
- Do you have any plans to talk with (name) / the stroke organisation again about support for you?
- What kind of support did you expect when you accepted a [stroke organisation] visit?
- Did it meet your expectations?
- Overall, what were your impressions of the visit?
- Are there any practical ways that the [stroke organisation] has influenced your own life as a caregiver? [Or have you found your own means of support, without reference to the stroke organisation?]

Second interviews: Questions about perceptions of impact support

[Last time we spoke] You told me about the things that were suggested that might be of help. Did you try any of these things?

- If no, why not?

If yes :

- Did they help? Have you continued to use this support?
  - Why/why not?
  - Has it lead to other things that have helped you as a carer?
- Is there any support you would have liked but felt like you didn't get?
  - Why? / how do you think it would have helped?
  - Has that changed over time?

For peer review only

- Overall - How did you find the [stroke organisation] in terms of supporting you as a carer? [Was it helpful/unhelpful?] Explore more:
    - Did it make any difference to you as a carer? If yes - in what way?
    - Has the support you received made any difference to the person you care for?
    - Is there anything you would have liked to have been done differently?

Finally

- Is there anything else you would like to tell us about the support you received / support for carers that we haven't covered?

For peer review only

SUPPLEMENTARY MATERIAL 2

Topic guides for staff

Frontline staff topic guide

Current practice

- Could you tell me briefly about your role [core tasks/expectations] within the stroke organisation? (how long?)
  - Could you tell me about your role in relation to supporting carers?
- Could you talk me through your current process of supporting carers?
  - Identifying (and defining) carers
  - Initial contact (face to face/ by phone; planned or unplanned? Always with stroke survivor or alone – and how does this happen?)
  - Identifying needs (paperwork; recording information? Action plan / follow up?)
  - Are there any other things that help you support carers? (e.g. local services)
  - Anything that prevents you from supporting carers?
- How have you found the OSCARSS recruitment process?

Changes to current practice

- Apart from OSCARSS are there any other factors that have impacted on how you support carers?
  - Has the CSNAT-Stroke been discussed within or/and across services? (e.g. through networking/learning events) – **control arm, skip to final question**

Intervention arm only:

- Have you been using CSNAT-Stroke during your visits that have involved carers? (If no – reasons why not? If yes – how did you use it? How have you found using it?)
- How would you describe CSNAT-Stroke?
- Similarities or differences of CSNAT-Stroke to how you've worked with carers before? If no difference – why is this?
- Any occasions where you would use an alternative process to address carer needs?
  - Any adaptation of CSNAT-Stroke or deliberately doing something else? Why?

Mechanisms that have supported changes

- Can you tell me about anything that has supported your use and understanding of CSNAT-Stroke
- Has there been anything that has hindered your use of CSNAT-Stroke? What could have helped overcome these hindrances?

Outcomes

- Through using CSNAT-Stroke do you feel like this has had any impact on your work with carers? Any impact on your work with stroke survivors?
- Do you think you would you continue to follow CSNAT-Stroke in your work with carers (why/why not)? What would you need to support the continued use of CSNAT-Stroke (on an individual and organisational level)?
- What is your overall opinion of CSNAT-Stroke?
- Is there anything else you would like to tell us about how you support carers that we haven't covered?

For peer review only

Managers and Senior Leaders: Topic guide

Current practice

- Could you tell me about your role within the organisation? (Involvement in supporting carers/ knowledge of how carers are supported?)
- How important do you feel supporting carers is within the organisation?
- What is needed to provide carer support? Who in the organisation should provide carer support?

Changes to current practice

- How much do you know about CSNAT-Stroke? (similarities or differences to standard practice etc.)
- How did you find out about CSNAT-Stroke?

Mechanisms that have supported changes

- Do you know of anything that has helped or hindered the use of CSNAT-Stroke?
- What structures would need to be in place to support the use of an approach like CSNAT-Stroke?

Outcomes

- Has the use of CSNAT-Stroke within the organisation created any changes?
    - Anything else that might have contributed to these changes?
- Do you think that the use of CSNAT-Stroke will be continued to support carers (why/why not)?
    - Does it feel like part of the organisation's approach to supporting carers now?
    - What would the organisation need to support the continued use of CSNAT-Stroke?
    - Who would be best placed/resourced to provide the training for CSNAT-Stroke?
- What is your overall opinion of CSNAT-Stroke?
- Is there anything else you would like to tell us about how carers are supported or CSNAT-Stroke that we haven't covered?

For peer review only

SUPPLEMENTARY MATERIAL 3

T2 and T3 questionnaire items based on NoMAD for frontline staff in the intervention arm only

	T2 questionnaire (N=20)			T3 questionnaire (N=20)		
**Item**	**Agree *n***	**Neutral *n***	**Disagree *n***	**Agree *n***	**Neutral *n***	**Disagree *n***
**Coherence**						
I can see how the CSNAT Stroke differs from usual ways of working	14	4	2	11	8	1
Staff in this service have a shared understanding of the purpose of the CSNAT-Stroke	13	7	0	10	7	3
I can see the potential value of the CSNAT-Stroke for my work	17	0	3	18	2	0
I understand how the CSNAT-Stroke affects the nature of my own work	18	2	0	17	2	1
**Cognitive participation**						
I believe that participating in the CSNAT-Stroke is a legitimate part of my role	15	3	2	16	4	0
There are key people in the Stroke Association who drive the CSNAT Stroke forward and get others involved	5	11	4	5	12	3
I'm open to working with colleagues in new ways to use the CSNAT-Stroke	16	2	2	16	4	0
**Collective action**						
I can easily integrate the CSNAT-Stroke into my working practice.	14	1	5	13	6	1
Sufficient resources are available to support the CSNAT Stroke	11	3	6	13	3	4
Sufficient training is provided to enable staff in this service to implement the CSNAT-Stroke	14	2	4	17	3	0
I have confidence in other people's ability to use the CSNAT-Stroke	10	10	0	11	9	0
Work is assigned to those with skills appropriate to the CSNAT-Stroke	5	13	2	7	12	1
Management adequately supports the CSNAT-Stroke	1	13	6	10	7	3
**Reflexive monitoring**						
Staff in this service agree that the CSNAT-Stroke is worthwhile	13	5	2	13	7	0
I value the effects that the CSNAT-Stroke has had on my work	12	5	3	15	5	0
Feedback about the CSNAT-Stroke can be used to improve it in the future	16	4	0	20	0	0

For peer review only

I can modify how I work with the CSNAT-Stroke	16	1	3	13	6	1

For peer review only

CHALLENGES IMPLEMENTING A CARER SUPPORT INTERVENTION WITHIN A NATIONAL STROKE ORGANISATION: FINDINGS FROM THE PROCESS EVALUATION OF THE OSCARSS TRIAL

Consolidated criteria for reporting qualitative studies (COREQ): 32-item checklist

No	Item	Guide questions/description	Our response
**Domain 1: Research team and reflexivity**			
**Personal Characteristics**			
1	Interviewer/facilitator	Which authors conducted the interviews	SD conducted all the staff interviews, KWN, EP and CM conducted the carer interviews.
2	Credentials	What were the researcher's credentials? E.g. PhD, MD	SD, EP and CM have PhDs. KWN has an MSc.
3	Occupation	What was their occupation at the time of the study?	SD was Research Associate. EP and CM were Research Fellows. KWN was Research Project Manager.
4	Gender	Was the researcher male or female?	Researchers were all female
5	Experience and training	What experience or training did the researcher have?	All researchers had experience and training in qualitative research methods
**Relationship with participants**			
6	Relationship established	Was a relationship established prior to study commencement?	SD had no relationship established with staff interviewed prior to study commencement. KWN, EP and CM had no relationship established with carers interviewed prior to study commencement.
7	Participant knowledge of the interviewer	What did the participants know about the researcher? e.g. personal goals, reasons for doing the research	All participants were given Participant Information Sheets which explained the

For peer review only

			research and the researchers' role in the study.
8	Interviewer characteristics	What characteristics were reported about the interviewer/facilitator? e.g. Bias, assumptions, reasons and interests in the research topic	There are no relevant characteristics to report about the interviewers.
**Domain 2: study design**			
**Theoretical framework**			
9	Methodological orientation and Theory	What methodological orientation was stated to underpin the study? e.g. grounded theory, discourse analysis, ethnography, phenomenology, content analysis	Process evaluation informed by Normalisation Process Theory (NPT).
**Participant selection**			
10	Sampling	How were participants selected?	Purposive sample
11	Method of approach	How were participants approached? e.g. face-to-face, telephone, mail, email	Staff were approached by email, carers were approached by telephone if they gave consent when recruited to the trial.
12	Sample size	How many participants were in the study?	21 carers and 31 staff were interviewed. We received 140 responses to a staff questionnaire at 3 different time points.
13	Non-participation	How many people refused to participate or dropped out? Reasons?	13 staff members chose not to be interviewed for reasons including they did not have time or felt they had nothing to say in an interview. 11 carers did not participate in second interviews, mainly because they felt they had nothing more to add since their first interview.
**Setting**			

For peer review only - http://bmjopen.bmj.com/site/about/guidelines.xhtml

14	Setting of data collection	Where was the data collected? e.g. home, clinic, workplace	Over the telephone for all carers and most staff, and in the workplace for some staff.
15	Presence of non-participants	Was anyone else present besides the participants and researchers?	No
16	Description of sample	What are the important characteristics of the sample? e.g. demographic data, date	Carers and staff members in the stroke organisation who support carers as part of their role.
**Data collection**			
17	Interview guide	Were questions, prompts, guides provided by the authors? Was it pilot tested?	Interviews were semi-structured (questions and prompts included in supplementary material). No pilot testing took place.
18	Repeat interviews	Were repeat interviews carried out? If yes, how many?	Repeat interviews were carried out with 11 carers.
19	Audio/visual recording	Did the research use audio or visual recording to collect the data?	All interviews were audio recorded
20	Field notes	Were field notes made during and/or after the interview or focus group?	Field notes were made after each interview
21	Duration	What was the duration of the interviews or focus group?	30-60 minutes
22	Data saturation	Was data saturation discussed?	Yes by the researchers involved in analysis on an ongoing basis
23	Transcripts returned	Were transcripts returned to participants for comment and/or correction?	No, but the content of interviews were summarised at the end of each interview and checked with participants.
**Domain 3: analysis and findings**			
**Data analysis**			
24	Number of data coders	How many data coders coded the data?	5
25	Description of the coding tree	Did authors provide a description of the coding tree?	Yes this was based on the NPT framework

For peer review only

26	Derivation of themes	Were themes identified in advance or derived from the data?	Analysis was both deductive using NPT and deductive drawing on the data
27	Software	What software, if applicable, was used to manage the data?	Nvivo
28	Participant checking	Did participants provide feedback on the findings?	Some staff participants gave feedback on the findings. Our Research User Group of carers also provided feedback.
29	Quotations presented	Were participant quotations presented to illustrate the themes / findings? Was each quotation identified? e.g. participant number	Participant quotations are presented and identified.
30	Data and findings consistent	Was there consistency between the data presented and the findings?	Yes
31	Clarity of major themes	Were major themes clearly presented in the findings?	Yes
32	Clarity of minor themes	Is there a description of diverse cases or discussion of minor themes?	We were restricted by the word count so have focused on major themes in this paper.

For peer review only

BMJ Open

CHALLENGES IMPLEMENTING A CARER SUPPORT INTERVENTION WITHIN A NATIONAL STROKE ORGANISATION: FINDINGS FROM THE PROCESS EVALUATION OF THE OSCARSS TRIAL

Journal:	*BMJ Open*
Manuscript ID	bmjopen-2020-038129.R2
Article Type:	Original research
Date Submitted by the Author:	02-Nov-2020

Complete List of Authors:	Darley, Sarah; The University of Manchester, Alliance Manchester Business School<br>Knowles, Sarah; The University of Manchester, Alliance Manchester Business School<br>Woodward-Nutt, Kate; NIHR Collaboration for Leadership in Applied Health Research and Care, Greater Manchester<br>Mitchell, Claire; The University of Manchester, Manchester Academic Health Sciences Centre (MAHSC), Division of Neuroscience and Experimental Psychology<br>Grande, Gunn; The University of Manchester, Manchester Academic Health Sciences Centre (MAHSC), Division of Nursing, Midwifery and Social Work<br>Ewing, Gail; University of Cambridge, Centre for Family Research<br>Rhodes, Sarah; The University of Manchester, Manchester Academic Health Sciences Centre (MAHSC), Centre for Biostatistics, Division of Population Health Services Research and Primary Care, School of Health Sciences:<br>Bowen, Audrey; The University of Manchester, Manchester Academic Health Sciences Centre (MAHSC), Division of Neuroscience and Experimental Psychology<br>Patchwood, Emma; The University of Manchester, Manchester Academic Health Sciences Centre (MAHSC), Division of Neuroscience and Experimental Psychology

**Primary Subject Heading**:	Qualitative research
Secondary Subject Heading:	Public health, Health services research
Keywords:	Stroke < NEUROLOGY, QUALITATIVE RESEARCH, PUBLIC HEALTH

SCHOLARONE™ Manuscripts

BMJ

I, the Submitting Author has the right to grant and does grant on behalf of all authors of the Work (as defined in the below author licence), an exclusive licence and/or a non-exclusive licence for contributions from authors who are: i) UK Crown employees; ii) where BMJ has agreed a CC-BY licence shall apply, and/or iii) in accordance with the terms applicable for US Federal Government officers or employees acting as part of their official duties; on a worldwide, perpetual, irrevocable, royalty-free basis to BMJ Publishing Group Ltd ("BMJ") its licensees and where the relevant Journal is co-owned by BMJ to the co-owners of the Journal, to publish the Work in this journal and any other BMJ products and to exploit all rights, as set out in our licence.

The Submitting Author accepts and understands that any supply made under these terms is made by BMJ to the Submitting Author unless you are acting as an employee on behalf of your employer or a postgraduate student of an affiliated institution which is paying any applicable article publishing charge ("APC") for Open Access articles. Where the Submitting Author wishes to make the Work available on an Open Access basis (and intends to pay the relevant APC), the terms of reuse of such Open Access shall be governed by a Creative Commons licence – details of these licences and which Creative Commons licence will apply to this Work are set out in our licence referred to above.

Other than as permitted in any relevant BMJ Author's Self Archiving Policies, I confirm this Work has not been accepted for publication elsewhere, is not being considered for publication elsewhere and does not duplicate material already published. I confirm all authors consent to publication of this Work and authorise the granting of this licence.

For peer review only

TITLE PAGE

CHALLENGES IMPLEMENTING A CARER SUPPORT INTERVENTION WITHIN A NATIONAL STROKE ORGANISATION: FINDINGS FROM THE PROCESS EVALUATION OF THE OSCARSS TRIAL

Corresponding author
Name: Dr Sarah Darley
Address: Suite 1 Williamson Building, Centre for Primary Care and Health Services Research, Faculty of Biology, Medicine and Health, University of Manchester, Oxford Road, Manchester M13 9QQ, UK
Email: Sarah.Darley@manchester.ac.uk
Telephone: +44 161 275 7687

Authors: Sarah Darley, Sarah Knowles, Kate Woodward-Nutt, Claire Mitchell, Gunn Grande, Gail Ewing, Sarah Rhodes, Audrey Bowen, and Emma Patchwood on behalf of the OSCARSS Research Team

Author Affiliations

University of Manchester, Alliance Manchester Business School: Sarah Knowles PhD, Sarah Darley PhD

University of Manchester, Manchester Academic Health Sciences Centre (MAHSC), Manchester, UK:

- Division of Neuroscience and Experimental Psychology, School of Biological Sciences: Emma Patchwood PhD, Prof Audrey Bowen PhD, Claire Mitchell, PhD
- Division of Nursing, Midwifery and Social Work, School of Health Sciences: Prof Gunn Grande PhD
- Centre for Biostatistics, Division of Population Health Services Research and Primary Care, School of Health Sciences: Sarah Rhodes MSc

University of Cambridge, Centre for Family Research, Cambridge, UK: Gail Ewing PhD

National Institute for Health Research (NIHR) Collaboration for Leadership in Applied Health Research and Care Greater, Manchester (CLAHRC GM): Kate Woodward-Nutt MSc

Manuscript word count (excluding title page, abstract, references, figures and tables): 4626
Abstract word count: 296
Tables: 3
Figures: 1

For peer review only

KEYWORDS
Stroke, Caregivers, Normalisation Process Theory, Process evaluation

ABSTRACT

Objectives
To examine the implementation of an intervention to support informal caregivers and to help understand findings from the Organising Support for Carers of Stroke Survivors (OSCARSS) cluster randomised controlled trial (cRCT).

Design
Longitudinal process evaluation using mixed methods. Normalisation Process Theory informed data collection and provided a sensitising framework for analysis.

Setting
Specialist stroke support services delivered primarily in the homes of informal carers of stroke survivors.

Participants
OSCARSS cRCT participants including carers, staff, managers and senior leaders.

Intervention
The Carer Support Needs Assessment Tool for Stroke (CSNAT-Stroke) intervention is a staff-facilitated, carer-led approach to help identify, prioritise, and address support needs.

Results
We conducted qualitative interviews with: OSCARSS cRCT carer participants (11 intervention, 10 control), staff (12 intervention, 8 control), and managers and senior leaders (11); and obtained 140 responses to an online staff survey over three separate time points. Both individual (carer/staff) and organisational factors impacted implementation of the CSNAT-Stroke intervention and how it was received by carers. We identified four themes: staff understanding, carer participation, implementation, and learning and support. Staff valued the idea of a structured approach to supporting carers, but key elements of the intervention were not routinely delivered. Carers did not necessarily identify as “carers”, which made it difficult for staff to engage them in the intervention. Despite organisational enthusiasm for OSCARSS, staff in the intervention arm perceived support and training for implementation of CSNAT-Stroke as delivered primarily by the research team, with few opportunities for shared learning across the organisation.

Conclusions
We identified challenges across carer, staff, and organisation levels that help explain the OSCARSS cRCT outcome. Ensuring training is translated into practice and ongoing organisational support would be required for full implementation of this type of intervention, with emphasis on the carer-led aspects, including supporting carer self-identification.

Trial registration
OSCARSS cRCT ISRCTN58414120

ARTICLE SUMMARY

Strengths and limitations of this study

- Conducting a longitudinal process evaluation over the period of three years enabled us to ascertain how the intervention was implemented in practice and provide understanding of the neutral outcome of the trial.
- Mixed methods helped us capture the complexity of the intervention and allowed us to triangulate quantitative and qualitative data to increase validity and credibility of findings.
- We captured a range of perspectives from all participants in the trial, including staff implementing the intervention, carers receiving the intervention, senior managers and leaders within the organisation, and carers and staff within the control arm.
- Observation of implementation was not possible for practical reasons, which made intervention fidelity difficult to assess.

For peer review only

- We ran the process evaluation largely independent of the trial in order to establish effectiveness of the intervention under real world conditions. However, this meant that process information was not shared with the organisation until towards the end of the trial

FUNDING STATEMENT

This project was supported by the National Institute for Health Research Collaboration for Leadership in Applied Health Research and Care (NIHR CLAHRC) Greater Manchester, grant number [N/A] and Stroke Association, grant number [N/A]. NIHR had no role in study design, data collection, data analysis, data interpretation, or writing of the paper. Stroke Association partnered with NIHR in funding this study and was the specialist stroke service provider in OSCARSS. Some Stroke Association staff were research participants in this study and others contributed to discussions about findings and dissemination. The first author had full access to all the study data and had final responsibility for the decision to submit for publication. The views expressed in this article are those of the author(s) and not necessarily those of the NIHR, the Department of Health and Social Care, or the Stroke Association.

COMPETING INTERESTS

Dr. Grande reports grants from NIHR CLAHRC, during the conduct of the study; In addition, Dr. Grande has a patent Copyright issued.
Dr. Ewing reports grants from NIHR, during the conduct of the study; In addition, Dr. Ewing has a patent Copyright issued.
Mrs. Rhodes reports grants from NIHR, during the conduct of the study.
Prof. Bowen reports grants from NIHR, grants from Stroke Association, during the conduct of the study; grants from Stroke Association, grants from NIHR, outside the submitted work.
Dr. Patchwood reports grants from Stroke Association, outside the submitted work.
Dr Darley, Dr Knowles, Ms Woodward-Nutt and Dr Mitchell have nothing to disclose.

INTRODUCTION

Background

Informal caregivers, such as family members, play a significant role in providing daily support to stroke survivors who experience a range of life-limiting difficulties [1]. The caring role can be rewarding [2] but is also challenging with significant health implications [3], impacting on physical and emotional well-being, financial security, professional and social activities [4]. Current support for carers differs according to location but generally comes from hospital stroke units, local authority, specialist carer organisations, and stroke organisations [5].

Supporting informal carers is a statutory priority [6, 7, 8] but there is little clarity on how best to identify and assess the needs of people caring informally for stroke survivors [9, 10, 11]. One relevant approach is the Carer Support Needs Assessment Tool (CSNAT) intervention [12]: a comprehensive evidence-based assessment tool integrated within a multi-stage person-centred approach to individualised support. It was developed, implemented and tested in the context of palliative care with positive outcomes [13, 14, 15]. As one of the few available evidence based interventions for carers that are carer-led and that enable comprehensive assessment and support, the CSNAT was considered worth exploring for supporting informal carers of people with long-term health conditions. For the present study, the CSNAT intervention was adapted for use in stroke care, through collaboration with a specialist stroke organisation and a group of lay carers. The involvement of carers in particular was integral in making the wording stroke specific and informing implementation of the intervention in practice [16]. The adaptation resulted in the CSNAT-Stroke intervention, including a staff training and implementation package tailored to the stroke organisation. Training was mainly delivered to frontline staff who are non-clinical support staff that provide personalised information, advice and support to stroke survivors and their carers In brief, the intervention is a person-centred, structured process of needs assessment and support that is practitioner-facilitated, but carer-led, comprising a four-stage process, including a needs assessment tool and action plan (see Table 1).

Table 1 CSNAT-Stroke intervention as intended in OSCARSS

Key details of the intervention using descriptors from the TIDieR checklist [17], adapted from Patchwood et al. [paper submitted]

Item	CSNAT-Stroke intervention, as intended for implementation within the stroke organisation
Why	To provide a person-centred process of assessment and support for carers that is practitioner-facilitated but carer-led and tailored to the carer's individual needs, which are likely to change over time. The intervention assumes that carers may have difficulty considering and expressing their needs.
What (materials and procedures)	Materials / procedures include: (A) four-stage process (components outlined below), (B) a needs assessment tool, (C) an action plan.<br><br>**1. Identifying and introducing:**<br><br>Staff identify the carer early and make clear that support is available ('scripts' for sensitive use of the term "carer"). Staff introduce needs assessment tool to carers during separate time with the carer, providing an opportunity to discuss support needs.<br><br>**2. Needs assessment**<br><br>Carers are given time to consider their support needs, self-completing the tool, identifying the areas in which they need more support and prioritising those most important to them. Staff normalise the practice of having separate time with the carer as well as stroke survivor if present, to support each as individuals.<br><br>**3. Assessment conversation and tailoring**<br><br>An assessment conversation between carer and stroke practitioners identifies the carer's individual support needs and what they feel would be most supportive within the domains of the needs assessment tool that they have prioritised. Support may be directly delivered by staff at this time (e.g. active listening, information, signposting/referrals) but helping carers identify sources of support they may wish to access themselves (self-help) or via family members and friends is also encouraged. Staff create service directories to support signposting/referral.<br><br>**4. Shared plan for action and review**<br><br>Carers record a plan of supportive input describing the actions taken or to be taken by the practitioner or carer to address identified needs, which will be subsequently reviewed as appropriate.<br><br>Each stage should be staff-facilitated but carer-led. At all stages the carer should be given the opportunity to say what is most important to them and what they feel would help support them.
Who provided	Frontline staff: Essential criteria for recruitment to this role includes GCSE education, experience providing care to vulnerable people, and good communication skills. Training in stroke and stroke-specific care is provided by the organisation. Additional intervention specific training provided to frontline staff in clusters allocated to intervention.<br><br>Training, co-delivered by the service provider and the research team, is with groups of staff over a half-day session involving instructional videos and scripts, role-play, and workbook completion.
How	After training the intervention should be implemented by staff during their routine support visits.
Where	Typically in carers' homes.
When and How much	The intervention should be used every time a staff member has contact with a carer and requires a minimum of 1 face-to-face support contact with the carer, with reviews likely.
Tailoring and modifications	Staff training is modified for those joining after the primary roll-out of the intervention and adapted to one-to-one delivery by the research team using video conferencing.

For peer review only

Organising Support for Carers of Stroke Survivors (OSCARSS) was a cluster randomised controlled trial (cRCT) which aimed to investigate the clinical- and cost-effectiveness of the CSNAT-Stroke intervention for supporting carers relative to a control of usual practice within the stroke organisation [18]. OSCARSS found no meaningful differences in self-reported clinical outcomes for carers, such as strain and distress, between intervention and control groups [paper submitted]. This paper presents findings from the embedded process evaluation of OSCARSS, which aimed to enrich understanding of the trial result, understand the factors influencing implementation and acceptability of a carer-led intervention, and consider implications for future research and practice.

METHODOLOGICAL APPROACH

Process evaluations provide insight into the 'black box' of implementation of a complex intervention [19] and help explain discrepancies between expected and observed outcomes within a context [20]. The process evaluation for OSCARSS used longitudinal mixed methods to examine ongoing experiences of and perspectives on both usual practice and the CSNAT-Stroke intervention from staff within the stroke organisation, as well as carers who receive their support and consented to join OSCARSS.

Normalisation Process Theory (NPT) was used as a framework to guide data collection and analysis, and sensitised us to certain points in the data for both agreement and resistance [21]. NPT is a commonly used framework in process evaluations of complex interventions in healthcare because it increases understanding of the dynamic processes involved during their implementation and helps to explain mechanisms that affect their outcomes [22]. NPT considers both agency (individual factors) and context (organisational factors) and proposes four constructs to explain how interventions are embedded into practice. Constructs include: Coherence (sense-making), Cognitive Participation (engagement and enrollment), Collective Action (how the work happens in existing systems), and Reflexive Monitoring (evaluation) [23].

SETTING AND SAMPLE

The OSCARSS cRCT ran from January 2017 to December 2018. It recruited 414 carers over an 18 month recruitment period from 35 randomised clusters made up of the stroke organisation's services across England and Northern Ireland (18 intervention and 17 control). Approximately 100 frontline staff who were employed at participating clusters during the study period were trained to participate in OSCARSS

The process evaluation purposefully sampled a wide range of participating frontline cluster staff and consented carers from both trial arms. Staff, managers and senior leaders were approached to participate by email; carers were approached by telephone if they consented to be contacted for this purpose during initial trial recruitment.

METHODS

We used mixed methods including training observations, qualitative interviews, and online questionnaires at different time points to capture the perspectives of different stakeholders during the course of the study (Figure 1). Triangulating methods and incorporating multiple perspectives and experiences aimed to increase the credibility and trustworthiness of our findings [24]. Ethics approval was obtained from the North West Lancaster Research Ethics Committee (ref: 16/NW/0657) and University of Manchester (ref: AMBS-2016-22). All participants gave informed consent.

Qualitative semi-structured interviews were conducted to provide rich descriptions of experiences and perspectives of both carers and staff. We conducted telephone interviews with carers as soon as possible after joining the main trial. Carer interviews were completed by members of the trial team (EP, KWN) and an experienced qualitative researcher (CM) between May 2017 and June 2018. Each carer was interviewed at the point of study consent and, if they agreed, again after primary outcomes had been collected at three months. Topic guides (supplementary material 1) were informed by the research aim and feedback from the RUG, and explored carers' experiences of support and their perceptions of the impact of support received.

Staff interviews were conducted with cluster frontline staff, managers and senior leaders between August and November 2018. All staff interviews were conducted by SD who had not been involved with staff training or support. Staff interviews were conducted face-to-face or over the telephone, depending on staff location and preference. Interview topic guides (supplementary material 2) were informed by NPT and aimed to capture

For peer review only

context and carer support practices, and strategic organisational priorities (relevant for managers and senior leadership). For intervention-allocated frontline staff, additional questions explored changes to practice, mechanisms that supported changes, and perceived outcome of changes to practice. Researchers made field notes and reflections after all interviews, which lasted between 30-60 minutes. Interviews were audio recorded with anonymised transcripts, field notes, and reflections uploaded to Nvivo software to facilitate thematic analysis.

Online questionnaires were distributed via email to all frontline staff involved in OSCARSS at three separate time points: T1 pre-randomisation of clusters in August 2016, T2 and T3 post-randomisation in August 2017 and July 2018 respectively. Questionnaires asked about existing practice, attitudes towards supporting carers, the wider context within which staff worked, and demographic information. At T2 and T3, the questionnaire for intervention arm staff additionally included items based on the NOrmalization MeAsure Development (NoMAD) [25] (supplementary material 3). NoMAD contains Likert items designed to explore intervention implementation, based on NPT.

Staff training in the intervention arm was typically delivered by the stroke organisation over half day group sessions and observed by KWN and EP. Field notes were taken to capture: perceived engagement of attendees with the training; whether all components of the training had been delivered as intended; types of questions asked by attendees and responses given. Staff also completed training feedback forms to explore their understanding of key components of the intervention.

Patient and public involvement

A study specific Research User Group (RUG) of ten individuals with experience of caring for a stroke survivor was set up in December 2015, at the planning stages of OSCARSS. Through regular meetings (2015-2019) and representation on the Trial Management Group the priorities, experiences, and preferences of the RUG informed development of the research questions and the design, analysis, and dissemination of both the trial and process evaluation. The RUG advised on participant recruitment and were central in limiting the burden of participation for carers. The RUG also supported adaptation of the CSNAT intervention and staff training package, including role-playing videos of the intervention in practice. A video summarising their role in OSCARSS is available on the study website [https://www.arc-gm.nihr.ac.uk/projects/oscarss] and a paper reflecting on their input [16]. In the early stages of data analysis three meetings were held to focus on the process evaluation where SD, SK and EP shared and discussed themes from the data with the RUG. In these meetings, NPT and the value of this framework for understanding the findings were collaboratively discussed. The RUG helped us consider rival explanations, new ideas, and implications on practice.

DATA ANALYSIS

Qualitative data

Several members of the research team (CM, EP, KWN, SD) used an inductive approach to analyse the interview data. This involved repeated detailed readings of all transcripts to identify dominant and frequent aspects relevant to the research aim that were developed into a coding framework. This framework was further developed by process evaluation leads (SD and SK) who took a deductive approach to the analysis by applying the NPT framework to the findings. This approach enabled the existing codes to be organised under the four NPT constructs. Analysis was an iterative process and the combination of inductive and deductive approaches allowed codes to fit into a structure, relate to each other in meaningful, study-important ways [26] and enabled identification of prominent themes. Other relevant data (e.g. training materials, observation field notes, and staff training feedback questionnaires) were reviewed throughout analysis (by SD and SK) to provide further context.

Quantitative data

Questionnaire data were analysed descriptively to provide summary information on usual practice of supporting carers within the stroke organisation and included NoMAD items relating to use and acceptability of the intervention.

Data synthesis

All data were synthesised and thematically analysed using the four main constructs of NPT [23] to identify patterns across the data [27]. As in previous studies this process allowed us to combine qualitative and quantitative findings [28] into one explanatory framework [26]. Researchers experienced in NPT (SD and SK) led this method of data analysis following a continuous, iterative process of reviewing and refining themes through discussions with the research team and RUG. We analysed at two levels: the individual and the organisation.

For peer review only - http://bmjopen.bmj.com/site/about/guidelines.xhtml

Draft findings were presented to multiple audiences, including internal PCPI collaborators (the RUG), Trial Management teams, and at external conferences and seminars. This enabled input from a multiplicity of viewpoints and supported reflective discussions to reach consensus on core, prominent themes [24].

RESULTS

Participants

Twenty-one carers of stroke survivors participated in first interviews (11 intervention, 10 control) and 11 agreed to follow up interviews (4 intervention, 7 control) (Table 2). Carers typically declined interviews when they felt they had nothing new to add.

Table 2 Carer characteristics

Key

SS needs score = independence level as reported by Carer (Min score = 1, Max score= 3)
C= control arm, I= intervention arm
M = Male, F = Female

Allocation	Gender	Age	Relation to Stroke Survivor	Stroke date	Ethnicity	SS needs score	Days from consent to interview	Status (1st/2nd interviews)
I	M	50-59	Partner	Feb 2017	White	2	33	completed 1&2
I	F	50-59	Daughter	May 2017	White	2.7	28	completed 1&2
I	M	60-69	Partner	May 2017	Asian	3	10	completed 1&2
I	F	60-69	Partner	Jan 2017	White	2.7	18	completed 1&2
I	F	30-39	Daughter	Jan 2017	Asian	1.7	4	opted out 2
I	F	50-59	Daughter	Aug 2017	White	3	21	opted out 2
I	F	50-59	Partner	June 2017	White	1	3	opted out 2
I	F	70-79	Partner	March 2017	White	1.7	19	opted out 2
I	M	70-79	Friend	March 2018	White	2	28	opted out 2
I	M	70-79	Partner	June 2017	White	1	29	opted out 2
I	F	70-79	Partner	Nov 2017	White other	1	65	opted out 2
C	F	40-49	Partner	Nov 2017	White	1	16	completed 1&2
C	F	60-69	Partner	Jan 2017	White	3	29	completed 1&2
C	M	60-69	Partner	Feb 2017	White	1.8	20	completed 1&2
C	M	60-69	Partner	Nov 2017	White	2.7	23	completed 1&2
C	F	60-69	Partner	Jan 2018	White	1.8	17	completed 1&2
C	F	70-79	Partner	Feb 2018	White	2.2	29	completed 1&2
C	F	70-79	Partner	March 2017	White	3	14	completed 1&2
C	F	60-69	Parent	July 2016	White	1.8	20	opted out 2
C	F	50-59	Daughter	Sep 2017	White	2.7	77	opted out 2
C	F	50-59	Daughter	Dec 2017	White	1.6	46	opted out 2

Thirty-one staff members were interviewed (frontline staff: 12 intervention, 8 control; managers and senior leaders across clusters: 11). Thirteen additional frontline staff declined interviews. Staff participants were based

For peer review only

across England and Northern Ireland, included both full and part-time workers and length of service ranged from less than a year to 23 years.

We observed 25 hours of staff training on the CSNAT-Stroke intervention over six group sessions, including 47 intervention-allocated staff. Response rates to the online staff questionnaire were: T1 72% (62 pre-intervention), T2 48% (20 intervention, 21 control), T3 35% (20 intervention, 17 control). Due to staff turnover, the same staff did not necessarily answer each questionnaire. Questionnaire respondents were predominantly female, aged 43 and over, worked part-time, and length of service ranged from less than one year to over ten.

Themes

In this section, we draw upon four themes identified through the NPT framework to report factors that impacted on implementation of the intervention (Table 3). For this purpose we mainly present data from the staff intervention arm (interviews and questionnaires T2 and T3), as well as from carers, managers and senior leaders.

Table 3 Themes identified through the NPT framework

NPT construct and definition	Relevant questions considered to guide analysis	Theme from data
**Coherence:** Understanding and valuing of the intervention	Was the intervention understood as different from usual practice?<br>What were the beliefs and behaviours that defined the new approach?<br>Was there a shared understanding of the intervention?<br>Was there a benefit identified in following the intervention?	**Limited understanding of different components of the intervention:** Although all staff valued the ethos of a carer-led approach to supporting carers, despite the training staff demonstrated a limited understanding of the whole intervention.
**Cognitive Participation:** Engagement in and activation of the intervention by individuals and groups	How was the intervention initiated?<br>Did staff and carers feel like legitimate participants in the intervention?<br>How were staff and carers enrolled and engaged in the intervention?<br>What promoted or inhibited participation in the intervention?	**Limited carer participation in the intervention:** Staff engaged with the intervention but struggled to persuade carers to participate and carers did not necessarily see themselves as legitimate participants.
**Collective Action:** How participants work to carry out the intervention	How was the intervention carried out?<br>What were the beliefs and behaviours of staff and carers that define and organise the work?<br>How did staff fit the new approach into their existing practice and context?<br>Who was best placed to make use of the intervention?	**The intervention was not fully implemented:** Key components of the approach (such as a separate conversation between carer and staff member, and completion of the action plan) were not always implemented.
**Reflexive Monitoring:** How the intervention is appraised and evaluated	What factors promoted or inhibited appraisal of the intervention?<br>How was the intervention monitored and evaluated?<br>How did staff and carers appraise the practice?<br>Was the intervention modified or adapted?	**Limited opportunities to share learning and ongoing support for the intervention:** All staff positively appraised the intervention as they understood it, but there were limited opportunities to share learning and receive ongoing support from across the stroke organisation.

We present our themes below, which encompass both the individual staff and carer level, and the organisational level. Using the NPT framework to take both levels into account helps identify the individual behavioural changes and agency, as well as the organisational structural and cultural changes required to implement a complex intervention [22].

Understanding of the intervention

More than half of intervention arm staff respondents to T2 and T3 questionnaires said they could see how the intervention differed from their usual way of working and could be easily integrated into current practice. However, in the interviews where this issue was explored in detail there were mixed perceptions of the difference, particularly for staff who had been in the role for a number of years:

> "I'd say it was quite similar, it's just the way it's outlaid isn't it really? What I do is not on paper as such… I've got experience behind me and I've worked with carers for a long time." [Frontline staff member 2, intervention, interview]

The desire for a structured, formalised approach to offering carer support came through strongly from all staff, including senior leaders. Over half the intervention respondents to T2 and T3 agreed that staff in their service had a shared understanding of the intervention. However, the intervention was primarily perceived as solely the paper-based needs assessment tool:

> "It's been a real pleasure to use, I consider it useful …in the beginning it was like okay great, more paper, but actually it's one of my most useful pieces of paper and I've got a lot of paper." [Frontline staff member 3, intervention, interview]

The interviews helped identify the lack of **Coherence** of the intervention through descriptions by staff of not fully implementing it and the similar accounts of support received by carers in both trial arms. Staff also appeared to see certain parts of the intervention, such as the action plan, as duplicating existing practice rather than a route to empowering carers to take ownership of actions for themselves:

> "I have to confess, I didn't [use the action plan]… I would follow it up in a letter … I don't see the point in doing [the action plan] …I'd be duplicating it." [Frontline staff member 4, intervention, interview]

Frontline staff positively appraised the OSCARSS training and, in feedback forms, highly endorsed that they understood each stage of the intervention and felt confident to deliver it. Despite this, interviews suggested that the training and ongoing support had not been sufficient to equip staff with a full understanding of the carer-led process of the intervention.

Carer participation

Carers in both arms of the study reported doing a wide range of caring tasks including personal care, practical tasks, and financial administration. They described the impact of caring on their emotional and physical wellbeing, restricted social life, having to give up work, and financial implications:

> "I'm 77 and it is hard work… it doesn't switch off." [Carer 1, intervention, interview 1]

However, during support visits carers did not always feel able to consider their own needs as their priority was the person they were caring for. This inhibited their **Cognitive Participation** in the intervention. Frontline staff saw this as a persistent issue, despite training for the intervention that included how to introduce CSNAT-Stroke during initial contact in a way that was sensitive to carer self-identification:

> "Where there's sometimes debate is when you're labelling someone as a carer and often someone's wife, for example, would say - I'm not his carer, I'm his wife." [Frontline staff member 1, intervention, interview]

Carers in the control arm appraised the support they received from the stroke organisation just as positively as carers did in the intervention arm, particularly when they received little support from other organisations. Generally all carers interviewed said that they were grateful to have somebody interested in their needs and able to provide practical help.

Implementation of intervention

The initiation of a separate conversation with a carer to discuss their needs is an important part of the intervention that was championed by the RUG. Observation notes showed that this point was often debated in the group training sessions, with some staff anticipating difficulty in talking with carers separately to stroke survivors. Staff described that historically these separate conversations tended to be intermittent and initiated by

carers (often on the doorstep at the end of a visit), whereas OSCARSS training encouraged staff to routinely initiate a separate conversation and normalise this in advance of a visit. Whilst staff saw the value of this in principle, they did not always initiate it in practice and instead many reported asking carers for the first time about meeting separately during the visit and in front of the stroke survivor:

> "What I find is I'll introduce it but people don't want to go off and talk on their own. They want to stay with the stroke survivor. Some people do, but some people don't. They said, oh no, that's fine, we can talk quite openly with each other, so you can't make people go and talk on their own." [Frontline staff member 5, intervention. interview]

The lack of full implementation through **Collective Action** was also evident in the needs assessment process. Staff would often assume what carers' needs might be and anticipate not being able to meet them, which led to a reluctance to comprehensively discuss needs:

> "To sort of say, okay, I recognise you're struggling with that and I don't have a solution… you're perceiving them to think, what was the point of asking me if I needed help - you've just highlighted the fact I need help and there's no services to help me." [Frontline staff member 1, intervention, interview]

Frontline staff had a well-meaning, strong desire to practically meet carer needs and 'fix' problems. However, core tenets of the carer-led approach of the intervention were that needs should be heard to be normalised and permit collaborative problem-solving, tailoring support to the carer's priorities.

Despite the lack of full implementation, components of the intervention that were understood were positively appraised by individual staff. For example, many expressed a desire to continue using the needs assessment tool in their meetings with carers beyond the trial. Managers were also keen to continue a systematic, structured approach to supporting carers.

Shared learning and ongoing support

The focus on carers and the carer-led approach of the intervention aligned with organisational values and was strongly supported by senior leaders and managers. However, managers did not have a comprehensive understanding of the intervention and whilst frontline staff perception of management support for the intervention increased over time, they saw training for and communications about the intervention as coming from the research team rather than the organisation:

> "I don't think really the [organisation] got involved much 'cause it was separate, wasn't it? Some [services] were included, some weren't, so I think they just left [the research team] to it and let you support us." [Frontline staff member 2, intervention, interview]

Frontline staff were viewed as most appropriate to deliver the intervention, although over half of T2 and T3 respondents in the intervention arm were neutral on whether work was assigned to those with skills appropriate to the intervention. At T2 and T3 frontline staff expressed openness to working with colleagues in new ways to use the intervention and in interviews frontline staff expressed a desire to learn from or share experiences with other teams.

However, there appeared to be few opportunities for shared learning on the intervention. Staff usually worked from home and did not have regular contact with each other, so opportunities were unlikely to happen without managerial planning. **Reflexive Monitoring** was limited with no systematic monitoring or evaluation of the intervention within the organisation, although almost all of T2 and T3 intervention respondents agreed that feedback could be used to improve the intervention.

During OSCARSS, services across the organisation experienced significant reorganisation internal staffing changes and commissioning changes. In interviews some frontline staff described difficulty participating in the intervention within the context of internal and external changes and pressures:

> "OSCARSS hasn't been a priority at all, we've been going through a lot of changes, we've lost some of our funding, we've had commissioned services being cut. There has been much more priority than doing the trial." [Frontline staff member 6, intervention, interview]

Just over half T2 and T3 intervention respondents agreed or strongly agreed that sufficient resources were available to support the intervention. However, in staff interviews a lack of time for and during visits to stroke survivors and carers was highlighted as inhibiting participation in the intervention:

"I think time would be the main thing, because… a lot of [frontline staff] just wouldn't necessarily factor the time in to a visit because they're already quite long, and there is already quite a lot of things to do within that visit." [Senior Leader, interview]

DISCUSSION

The OSCARSS process evaluation helps us understand the context in which the intervention was delivered within the trial and provides some explanation of why no meaningful added benefit was evident when comparing intervention with usual practice. We identified significant challenges to successful implementation of the intervention at both individual and organisational levels. Frontline staff were mainly positive about the intervention, insofar as they wished to enhance carer support, but they did not have a full understanding of the *carer-led* aspects of the intervention. As in previous studies [29] this lack of coherence was a key factor in the intervention not being fully implemented. The training that frontline staff received through OSCARSS (half a day face-to-face or 1-3 hours over Skype) and ongoing informal support from the research team was not sufficient to provide this understanding. Frontline staff cared deeply about supporting carers, but their tendency to anticipate and 'fix' needs limited opportunities for the approach to be carer-led. Key stages of the intervention were also not routinely implemented, such as a separate conversation and completion of an action plan. Consistent with this finding, interviews with carers demonstrated little difference in support provided across the trial's intervention and control arms. Despite positive engagement and organisational enthusiasm for OSCARSS, the intervention was not delivered as intended. The findings demonstrate challenges to delivery of carer-led assessment and support, including overcoming the reluctance of carers to express their own needs, frontline staff anticipating carer needs, and the importance of ongoing organisational support and learning.

Although there is strong evidence for the need to support carers of stroke survivors [30, 31] and a statutory requirement to do so [8] those considering implementing carer-led interventions should be aware of the challenges and solutions highlighted by OSCARSS. Carers in our study described the range of tasks they were responsible for in providing care and the impact this has on their lives. However, in line with other studies the carers did not always feel ready to accept support for themselves and did not necessarily see themselves as "carers" [32, 33] and the intervention was not implemented in a way to address this issue. Using NPT emphasised that motivation and 'buy in' for an intervention relies on engagement of both practitioner and service recipient to 'enroll' themselves as eligible and appropriate for an intervention to work. This may be particularly challenging for carers. Our findings contribute to this often overlooked yet vital aspect in implementation theory [34] and our multi-stakeholder analysis enabled us to explore both individual and wider contextual factors that impact on implementation in practice.

Our process evaluation has shown the difficulty implementing a complex intervention that follows a carer-led ethos, despite strong organisational enthusiasm and staff willingness to support carers. Our findings show that staff would have welcomed additional ongoing support and shared learning opportunities to gain a full understanding of the carer-led approach. Support mechanisms used in previous CSNAT intervention implementations, such as developing staff champions [35, 36] may have ensured that managerial commitment was communicated to frontline staff. However this was difficult within the design of the cluster RCT as in some services a cluster may have only included one or two staff members. The trial team tried to offer similar support and championing to intervention arm frontline staff, but our findings indicate that such support would have been more effective coming from the organisation itself. Many carers across both trial arms said that frontline staff in the organisation were one of their main sources of support and this was highly valued. In OSCARSS, the intervention was developed to be used within the pragmatic organisational context of individual staff supporting both stroke survivor and carer, requiring no additional resources. However, our findings highlight the barriers for coordinators to fully support both individuals within their available resources and limited time. Our findings also highlight the particular complexity of meeting needs of carers who struggle to recognise their own needs, consideration of timing of support, and how to sensitively and appropriately facilitate conversations with carers and stroke survivors.

The process evaluation was designed and delivered by a dedicated evaluation team (SD, SK) alongside the main trial and used multiple methods over time to generate rich and in-depth data. It used NPT to bring together these data that provided a range of perspectives at different time points and sampled from both trial arms across clusters. Through examining implementation at the individual and organisational level, the process evaluation offers insight into why the intervention was not fully implemented and contributes to knowledge on implementing carer-led approaches in health care. A limitation to the study was that observation of the intervention in real-time was not conducted. Reasons for this included the difficulty of minimising Hawthorne effects on staff implementing the intervention, ethical considerations of carers discussing sensitive information at a potentially distressing time, and practicalities of observation within a large-scale study of 35 clusters. This limitation impacted on the extent to which we could assess fidelity as we were not able to triangulate interview and question data with observations of

practice. However, we recruited carers and staff from different geographical areas within the trial and triangulated multiple data to get an understanding of implementation from the perspective of several stakeholder groups. The process evaluation was also run largely independent of the trial in order to establish effectiveness of the intervention under real world conditions [20]. However, this resulted in sharing process information with the organisation only towards the end of the trial, meaning that additional strategies to help support full implementation of the intervention were not explored. In hindsight, taking into account our findings, it may have been useful to have planned the process evaluation in stages with an early review, and also run an initial feasibility study before the full trial.

The process evaluation showed that the intervention was not fully implemented as intended, therefore we cannot say whether it may provide a benefit to carers of stroke survivors. Using NPT we have highlighted areas that need to be addressed in future research of this intervention or similar person-led approaches to supporting carers of stroke survivors. Considerations include firstly, how to train and support staff including strategies to help with engaging carers reluctant to prioritise their own needs. Secondly, providing clear communication pathways to ensure adoption of the intervention, involving management goals top down to frontline staff and also bottom up enabling practitioners to raise implementation difficulties and have them resolved. Thirdly, having a network of staff champions as role models and for problem solving. Fourthly, clarity at the outset about resources needed for implementation and sustainability.

CONCLUSIONS

This paper shows the value of process evaluation in helping to interpret neutral trial results, such as OSCARSS. It demonstrates how both staff and service user perceptions and organisational demands can impede the delivery of key intervention components, even with significant buy-in to the need for the intervention itself. Our findings identified certain challenges, as well as opportunities, that contribute to knowledge on implementing carer-led support interventions. We recommend that future intervention work should concentrate on addressing organisational factors which can both help and hinder implementation and that all levels within the organisation are engaged, including undertaking training, to have a full understanding of all components of the intervention.

AUTHOR CONTRIBUTIONS

All authors (SD, SK, KWN, CM, GG, GE, SR, AB, EP) contributed to the design of this study. SD, KWN, CM and EP contributed to recruitment, collected the data and contributed to the analysis. All authors (SD, SK, KWN, CM, GG, GE, SR, AB, EP) contributed to interpretation of findings and writing of the manuscript.

ACKNOWLEDGEMENTS

The authors would like to thank all of the carers and staff members who participated and contributed to this study.

Jessica Stolberg contributed to collection of questionnaire data and Verity Longley contributed to analysis. The trial steering committee contributed to the study design and interpretation of findings. The study specific carer advisory research group contributed to the study design and interpretation of findings: Kelly Burke, Christine Halford, Natalie Halford, Geoff Heathcote, Kath Purcell and Ben Wright. We would like to thank members of the supportive NIHR CLARHC GM team who helped make the study possible including: Caroline O'Donnell (data analyst); Alison Littlewood and Katy Rothwell (programme managers); Sam Wilkinson, Amy Woodhouse and Rose Crees (administrative assistants); Aneela Macavoy and Zoe Ashton (research facilitators). We also wish to thank and acknowledge Stroke Association staff, including Chris Larkin, Elaine Roberts, Cass Saunders and Kathryn Staley.

DATA SHARING STATEMENT

All data relevant to the study are included in the article or uploaded as supplementary information. No additional data available.

REFERENCES

1 Crichton SL, Bray BD, McKevitt C, *et al.* Patient outcomes up to 15 years after stroke: survival, disability, quality of life, cognition and mental health. *J Neurol Neurosurg Psychiatry Res*, 2016;87:1091–1098.

For peer review only

2 Mackenzie A, Greenwood N. Positive experiences of caregiving in stroke: a systematic review. *Disabil Rehabil*. 2012;34(17):1413-1422.

3 Noonan MC, Wingham J, Taylor RS. 'Who Cares?' The experiences of caregivers of adults living with heart failure, chronic obstructive pulmonary disease and coronary artery disease: a mixed methods systematic review *BMJ Open,* 2018;8:e020927.

4 Quinn K, Murray C, Malone C. Spousal experiences of coping with and adapting to caregiving for a partner who has a stroke: a meta-synthesis of qualitative research. *J Disabil Rehabil,* 2014;36:185–198.

5 The Stroke Association. Stroke: a carer's guide. 2018. Available: https://www.stroke.org.uk/sites/default/files/media-root/f04_stroke_a_carers_guide_v2.2.pdf [accessed 18/06/2020]

6 Carers UK. NHS England's Long Term Plan and what it means for carers, 2019. Available: https://www.carersuk.org/for-professionals/policy/policy-library/the-nhs-long-term-plan-and-what-it-means-for-carers [accessed 12 August 2019]

7 Department of Health & Social Care. Carers Action Plan 2018 to 2020: Supporting carers today. Available www.nationalarchives.gov.uk/doc/open-government-licence/ [accessed 6 September 2019]

8 Department of Health, Care Act. 2014. Available: http://www.legislation.gov.uk/ukpga/2014/23/contents/enacted/data.htm [accessed 21 November 2019]

9 Ellis G, Mant J, Langhorne P. *et al.* Stroke liaison workers for stroke patients and carers: an individual patient data meta-analysis. *Cochrane Database Syst Rev.* 2010; 2010(5):CD005066.

10 Legg LA, Quinn TJ, Mahmood F. *et al.* Non-pharmacological interventions for caregivers of stroke survivors. *Cochrane Database Syst Rev*. 2011;(10):CD008179.

11 Forster A, Brown L, Smith J, *et al.* Information provision for stroke patients and their caregivers. *Cochrane Database Syst Rev*. 2012;11(11):CD001919.

12 Ewing G, Grande GA. Development of a Carer Support Needs Assessment Tool (CSNAT) for end-of-life care practice at home: *Palliat Med,* 2012;27:244-256.

13 Ewing G, Austin L, Grande G. The role of the Carer Support Needs Assessment Tool in palliative home care: A qualitative study of practitioners' perspectives of its impact and mechanisms of action. *Palliat Med*, 2016;30:392–400.

14 Ewing G, Brundle C, Payne S. The Carer Support Needs Assessment Tool (CSNAT) for Use in Palliative and End-of-life Care at Home: A Validation Study. *J Pain Symptom Manage*, 2013;46:395-405.

15 Aoun SM, Grande G, Howting D, *et al.* The impact of the carer support needs assessment tool (CSNAT) in community palliative care using a stepped wedge cluster trial, *PLoS One*, 2015;10:4.

16 Mitchell C, Burke K, Halford N, *et al.* Value and learning from carer involvement in a cluster randomised controlled trial and process evaluation - Organising Support for Carers of Stroke Survivors (OSCARSS). *Res Involv Engagem* 2020;6:21.

17. Hoffman TC, Glasziou PP, Boutron I, *et al.* Better reporting of interventions: template for intervention description and replication (TIDieR) checklist and guide, *BMJ*, 2014;348:g1687

18 Patchwood E, Rothwell K, Rhodes S, *et al.* Organising Support for Carers of Stroke Survivors (OSCARSS): study protocol for a cluster randomised controlled trial, including health economic analysis. *Trials*, 2019;20:19.

19 Grant A, Treweek S, Dreischulte T, *et al.* Process evaluations for cluster-randomised trials of complex interventions: a proposed framework for design and reporting. *Trials*, 2013;14:15.

20 Moore GF, Audrey S, Barker M, *et al.* Process evaluation of complex interventions: Medical Research Council guidance. *BMJ*, 2015;350:h1258.

21 Mills MR, Bettis PJ. Organizational Identity and Identification During a Departmental Reorganization. In: Anfara VA, Mertz NT, eds. Theoretical Frameworks in Qualitative Research. London: Sage 2006:73-84.

22 May CR, Cummings A, Girling M, *et al.* Using Normalization Process Theory in feasibility studies and process evaluations of complex healthcare interventions: a systematic review. *Implement Sci*, 2018;13:80.

For peer review only

23 May C, Finch T. Implementing, Embedding, and Integrating Practices: An Outline of Normalization Process Theory. *Sociology*, 2009;43:535–554.

24 Patton M. *Qualitative evaluation and research methods*. Beverly Hills, CA: Sage 1990.

25 May CR, Finch T, Ballini L, *et al.* Evaluating complex interventions and health technologies using normalization process theory: development of a simplified approach and web-enabled toolkit. *BMC Health Serv Res*, 2011;11:245

26 Moran-Ellis J, Alexander VD, Cronin A, *et al.* Triangulation and integration: Processes, claims and implications. *Qual Res*, 2006;6:45–59.

27 Braun V, Clarke V. Using thematic analysis in psychology, *Qual Res Psychol,* 2006;3:77-101.

28 Lewis NV, Dowrick A, Sohal A, *et al.* Implementation of the Identification and Referral to Improve Safety programme for patients with experience of domestic violence and abuse: A theory-based mixed-method process evaluation. *Health Soc Care Community*, 2019;27:e298-e312.

29 Lloyd A, Joseph-Williams N, Edwards A, *et al.* Patchy 'coherence': using normalization process theory to evaluate a multi-faceted shared decision making implementation program (MAGIC). *Implementation Sci*, 2013;8: 102

30 Haley WE, Roth DL, Hovater M, *et al.* Long-term impact of stroke on family caregiver well-being: a population-based case-control study. *Neurology*, 2015;84:1323–1329.

31 Kruithof WJ, Post MWM, Van Mierlo ML, *et al.* Caregiver burden and emotional problems in partners of stroke patients at two months and one year post-stroke: Determinants and prediction. *Patient Educ Couns*, 2016;99: 1632–1640.

32 Knowles S, Combs R, Kirk S, *et al.* Hidden caring, hidden carers? Exploring the experience of carers for people with long-term conditions. *Health Soc Care Community*, 2016;24:203–213.

33 Cameron JI, Gignac MAM. "Timing It Right": A conceptual framework for addressing the support needs of family caregivers to stroke survivors from the hospital to the home. *Patient Educ Couns*, 2008;70:305–314.

34 Kennedy A, Rogers A, Chew-Graham C, *et al.* Implementation of a self-management support approach (WISE) across a health system: a process evaluation explaining what did and did not work for organisations, clinicians and patients. *Implement Sci*, 2014;9:129.

35 Diffin J, Ewing G, Harvey G, *et al.* Facilitating successful implementation of a person-centred intervention to support family carers within palliative care: a qualitative study of the Carer Support Needs Assessment Tool (CSNAT) intervention. *BMC Palliat Care*, 2018;17:129.

36 Austin L, Ewing G, Grande, G. Factors influencing practitioner adoption of carer-led assessment in palliative homecare: A qualitative study of the use of the Carer Support Needs Assessment Tool (CSNAT), *PLoS One*, 2017;12:6.

For peer review only

FIGURES

Figure 1: Process evaluation methods

(Submitted as a separate file.)

This figure shows how the mixed methods used within the process evaluation were conducted alongside the cRCT

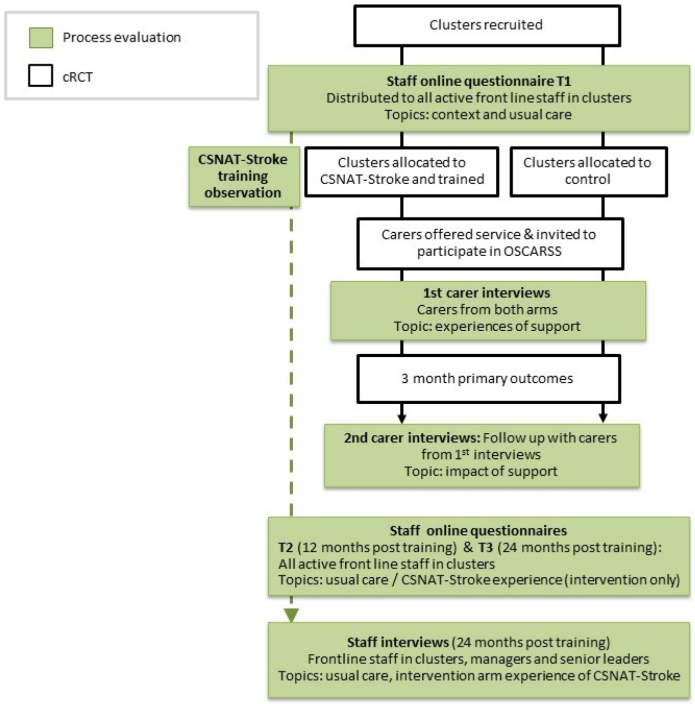

Figure 1 Process evaluation methods overview within the OSCARSS trial

254x190mm (96 x 96 DPI)

SUPPLEMENTARY MATERIAL 1

Topic guides for carer interviews

First interviews: Questions about experiences of support

I'd like to speak to you about contact you've had from [stroke organisation].

- Can you talk me through what happened during the visit?
  - Did you talk with X on your own or with others? (how was that / how did that go?)
  - Was any paperwork used? Possible additional probs : Can you tell me more about the paperwork you used? How was that / how did that go?
  - Did you keep a copy of any paperwork? If so, do you ever refer to it / find it helpful?
  - Did you talk about any support that you might need , as someone who is providing care/ support to <INSERT RELATIONSHIP eg mum >> How was that / How did that go?
  - Did you feel that you or the staff member 'took the lead' when talking? (Could re-visit 'paperwork' prompts here too)
- Did you come up with ideas of how to address the needs/issues you talked about? Can you talk me through them ?
  - Depending on if/how addressed earlier could repeat: Did you feel that you or the coordinator 'took the lead' when coming up with ideas?
  - Were you happy with the solutions/ ideas for support?
  - Were there any things that are not being addressed/ that it wasn't possible to address? What were these? How do you feel about this?
- Do you have any plans to talk with (name) / the stroke organisation again about support for you?
- What kind of support did you expect when you accepted a [stroke organisation] visit?
- Did it meet your expectations?
- Overall, what were your impressions of the visit?
- Are there any practical ways that the [stroke organisation] has influenced your own life as a caregiver? [Or have you found your own means of support, without reference to the stroke organisation?]

Second interviews: Questions about perceptions of impact support

[Last time we spoke] You told me about the things that were suggested that might be of help. Did you try any of these things?

- If no, why not?

If yes :

- Did they help? Have you continued to use this support?
  - Why/why not?
  - Has it lead to other things that have helped you as a carer?
- Is there any support you would have liked but felt like you didn't get?
  - Why? / how do you think it would have helped?
  - Has that changed over time?

For peer review only

- Overall - How did you find the [stroke organisation] in terms of supporting you as a carer? [Was it helpful/unhelpful?] Explore more:
    - Did it make any difference to you as a carer? If yes - in what way?
    - Has the support you received made any difference to the person you care for?
    - Is there anything you would have liked to have been done differently?

Finally

- Is there anything else you would like to tell us about the support you received / support for carers that we haven't covered?

For peer review only

SUPPLEMENTARY MATERIAL 2

Topic guides for staff

Frontline staff topic guide

Current practice

- Could you tell me briefly about your role [core tasks/expectations] within the stroke organisation? (how long?)
  - Could you tell me about your role in relation to supporting carers?
- Could you talk me through your current process of supporting carers?
  - Identifying (and defining) carers
  - Initial contact (face to face/ by phone; planned or unplanned? Always with stroke survivor or alone – and how does this happen?)
  - Identifying needs (paperwork; recording information? Action plan / follow up?)
  - Are there any other things that help you support carers? (e.g. local services)
  - Anything that prevents you from supporting carers?
- How have you found the OSCARSS recruitment process?

Changes to current practice

- Apart from OSCARSS are there any other factors that have impacted on how you support carers?
  - Has the CSNAT-Stroke been discussed within or/and across services? (e.g. through networking/learning events) – **control arm, skip to final question**

Intervention arm only:

- Have you been using CSNAT-Stroke during your visits that have involved carers? (If no – reasons why not? If yes – how did you use it? How have you found using it?)
- How would you describe CSNAT-Stroke?
- Similarities or differences of CSNAT-Stroke to how you've worked with carers before? If no difference – why is this?
- Any occasions where you would use an alternative process to address carer needs?
  - Any adaptation of CSNAT-Stroke or deliberately doing something else? Why?

Mechanisms that have supported changes

- Can you tell me about anything that has supported your use and understanding of CSNAT-Stroke
- Has there been anything that has hindered your use of CSNAT-Stroke? What could have helped overcome these hindrances?

Outcomes

- Through using CSNAT-Stroke do you feel like this has had any impact on your work with carers? Any impact on your work with stroke survivors?
- Do you think you would you continue to follow CSNAT-Stroke in your work with carers (why/why not)? What would you need to support the continued use of CSNAT-Stroke (on an individual and organisational level)?
- What is your overall opinion of CSNAT-Stroke?
- Is there anything else you would like to tell us about how you support carers that we haven't covered?

For peer review only

Managers and Senior Leaders: Topic guide

Current practice

- Could you tell me about your role within the organisation? (Involvement in supporting carers/ knowledge of how carers are supported?)
- How important do you feel supporting carers is within the organisation?
- What is needed to provide carer support? Who in the organisation should provide carer support?

Changes to current practice

- How much do you know about CSNAT-Stroke? (similarities or differences to standard practice etc.)
- How did you find out about CSNAT-Stroke?

Mechanisms that have supported changes

- Do you know of anything that has helped or hindered the use of CSNAT-Stroke?
- What structures would need to be in place to support the use of an approach like CSNAT-Stroke?

Outcomes

- Has the use of CSNAT-Stroke within the organisation created any changes?
    - Anything else that might have contributed to these changes?
- Do you think that the use of CSNAT-Stroke will be continued to support carers (why/why not)?
    - Does it feel like part of the organisation's approach to supporting carers now?
    - What would the organisation need to support the continued use of CSNAT-Stroke?
    - Who would be best placed/resourced to provide the training for CSNAT-Stroke?
- What is your overall opinion of CSNAT-Stroke?
- Is there anything else you would like to tell us about how carers are supported or CSNAT-Stroke that we haven't covered?

For peer review only

SUPPLEMENTARY MATERIAL 3

T2 and T3 questionnaire items based on NoMAD for frontline staff in the intervention arm only

	T2 questionnaire (N=20)			T3 questionnaire (N=20)		
**Item**	**Agree *n***	**Neutral *n***	**Disagree *n***	**Agree *n***	**Neutral *n***	**Disagree *n***
**Coherence**						
I can see how the CSNAT Stroke differs from usual ways of working	14	4	2	11	8	1
Staff in this service have a shared understanding of the purpose of the CSNAT-Stroke	13	7	0	10	7	3
I can see the potential value of the CSNAT-Stroke for my work	17	0	3	18	2	0
I understand how the CSNAT-Stroke affects the nature of my own work	18	2	0	17	2	1
**Cognitive participation**						
I believe that participating in the CSNAT-Stroke is a legitimate part of my role	15	3	2	16	4	0
There are key people in the Stroke Association who drive the CSNAT Stroke forward and get others involved	5	11	4	5	12	3
I'm open to working with colleagues in new ways to use the CSNAT-Stroke	16	2	2	16	4	0
**Collective action**						
I can easily integrate the CSNAT-Stroke into my working practice.	14	1	5	13	6	1
Sufficient resources are available to support the CSNAT Stroke	11	3	6	13	3	4
Sufficient training is provided to enable staff in this service to implement the CSNAT-Stroke	14	2	4	17	3	0
I have confidence in other people's ability to use the CSNAT-Stroke	10	10	0	11	9	0
Work is assigned to those with skills appropriate to the CSNAT-Stroke	5	13	2	7	12	1
Management adequately supports the CSNAT-Stroke	1	13	6	10	7	3
**Reflexive monitoring**						
Staff in this service agree that the CSNAT-Stroke is worthwhile	13	5	2	13	7	0
I value the effects that the CSNAT-Stroke has had on my work	12	5	3	15	5	0
Feedback about the CSNAT-Stroke can be used to improve it in the future	16	4	0	20	0	0

For peer review only

I can modify how I work with the CSNAT-Stroke	16	1	3	13	6	1

For peer review only

CHALLENGES IMPLEMENTING A CARER SUPPORT INTERVENTION WITHIN A NATIONAL STROKE ORGANISATION: FINDINGS FROM THE PROCESS EVALUATION OF THE OSCARSS TRIAL

Consolidated criteria for reporting qualitative studies (COREQ): 32-item checklist

No	Item	Guide questions/description	Our response
**Domain 1: Research team and reflexivity**			
**Personal Characteristics**			
1	Interviewer/facilitator	Which authors conducted the interviews	SD conducted all the staff interviews, KWN, EP and CM conducted the carer interviews.
2	Credentials	What were the researcher's credentials? E.g. PhD, MD	SD, EP and CM have PhDs. KWN has an MSc.
3	Occupation	What was their occupation at the time of the study?	SD was Research Associate. EP and CM were Research Fellows. KWN was Research Project Manager.
4	Gender	Was the researcher male or female?	Researchers were all female
5	Experience and training	What experience or training did the researcher have?	All researchers had experience and training in qualitative research methods
**Relationship with participants**			
6	Relationship established	Was a relationship established prior to study commencement?	SD had no relationship established with staff interviewed prior to study commencement. KWN, EP and CM had no relationship established with carers interviewed prior to study commencement.
7	Participant knowledge of the interviewer	What did the participants know about the researcher? e.g. personal goals, reasons for doing the research	All participants were given Participant Information Sheets which explained the

For peer review only

			research and the researchers' role in the study.
8	Interviewer characteristics	What characteristics were reported about the interviewer/facilitator? e.g. Bias, assumptions, reasons and interests in the research topic	There are no relevant characteristics to report about the interviewers.
**Domain 2: study design**			
**Theoretical framework**			
9	Methodological orientation and Theory	What methodological orientation was stated to underpin the study? e.g. grounded theory, discourse analysis, ethnography, phenomenology, content analysis	Process evaluation informed by Normalisation Process Theory (NPT).
**Participant selection**			
10	Sampling	How were participants selected?	Purposive sample
11	Method of approach	How were participants approached? e.g. face-to-face, telephone, mail, email	Staff were approached by email, carers were approached by telephone if they gave consent when recruited to the trial.
12	Sample size	How many participants were in the study?	21 carers and 31 staff were interviewed. We received 140 responses to a staff questionnaire at 3 different time points.
13	Non-participation	How many people refused to participate or dropped out? Reasons?	13 staff members chose not to be interviewed for reasons including they did not have time or felt they had nothing to say in an interview. 11 carers did not participate in second interviews, mainly because they felt they had nothing more to add since their first interview.
**Setting**			

For peer review only

14	Setting of data collection	Where was the data collected? e.g. home, clinic, workplace	Over the telephone for all carers and most staff, and in the workplace for some staff.
15	Presence of non-participants	Was anyone else present besides the participants and researchers?	No
16	Description of sample	What are the important characteristics of the sample? e.g. demographic data, date	Carers and staff members in the stroke organisation who support carers as part of their role.
**Data collection**			
17	Interview guide	Were questions, prompts, guides provided by the authors? Was it pilot tested?	Interviews were semi-structured (questions and prompts included in supplementary material). No pilot testing took place.
18	Repeat interviews	Were repeat interviews carried out? If yes, how many?	Repeat interviews were carried out with 11 carers.
19	Audio/visual recording	Did the research use audio or visual recording to collect the data?	All interviews were audio recorded
20	Field notes	Were field notes made during and/or after the interview or focus group?	Field notes were made after each interview
21	Duration	What was the duration of the interviews or focus group?	30-60 minutes
22	Data saturation	Was data saturation discussed?	Yes by the researchers involved in analysis on an ongoing basis
23	Transcripts returned	Were transcripts returned to participants for comment and/or correction?	No, but the content of interviews were summarised at the end of each interview and checked with participants.
**Domain 3: analysis and findings**			
**Data analysis**			
24	Number of data coders	How many data coders coded the data?	5
25	Description of the coding tree	Did authors provide a description of the coding tree?	Yes this was based on the NPT framework

For peer review only

26	Derivation of themes	Were themes identified in advance or derived from the data?	Analysis was both deductive using NPT and deductive drawing on the data
27	Software	What software, if applicable, was used to manage the data?	Nvivo
28	Participant checking	Did participants provide feedback on the findings?	Some staff participants gave feedback on the findings. Our Research User Group of carers also provided feedback.
29	Quotations presented	Were participant quotations presented to illustrate the themes / findings? Was each quotation identified? e.g. participant number	Participant quotations are presented and identified.
30	Data and findings consistent	Was there consistency between the data presented and the findings?	Yes
31	Clarity of major themes	Were major themes clearly presented in the findings?	Yes
32	Clarity of minor themes	Is there a description of diverse cases or discussion of minor themes?	We were restricted by the word count so have focused on major themes in this paper.

For peer review only
